# Altered G_i_ signaling in enteroendocrine K cells in vivo causes pronounced changes in glucose homeostasis

**DOI:** 10.1126/sciadv.aeb9805

**Published:** 2026-07-29

**Authors:** Osvaldo Rivera-Gonzalez, Liu Liu, Fiona M. Gribble, Frank Reimann, Jürgen Wess

**Affiliations:** ^1^ Molecular Signaling Section, Laboratory of Bioorganic Chemistry, National Institute of Diabetes and Digestive and Kidney Diseases, Bethesda, MD 20892, USA.; ^2^ MRC Metabolic Diseases Unit, Institute of Metabolic Science, University of Cambridge, Addenbrooke’s Hospital, Cambridge CB2 1TN, UK.

## Abstract

Glucose-dependent insulinotropic polypeptide (GIP) is an incretin hormone that promotes postprandial insulin release and euglycemia. Although GIP is the dominant incretin in humans, the precise mechanisms regulating GIP release from enteroendocrine K cells in vivo remain to be elucidated. Heterotrimeric G proteins of the G_i_ family regulate numerous key physiological functions. To explore the potential role of these proteins in modulating K cell function in vivo, we analyzed mutant mice that allowed us to selectively stimulate G_i_ signaling in K cells or studied mice lacking functional G_i_ in K cells only. Stimulation of K cell G_i_ signaling in vivo and in vitro led to reduced GIP release via an exchange protein directly activated by 3ʹ-5ʹ-cyclic adenosine monophosphate 2–dependent pathway and various metabolic deficits. In contrast, inactivation of this class of G proteins in K cells caused elevated plasma GIP levels and pronounced improvements in glucose homeostasis in lean and obese mice. These data provide important insights into the mechanisms of GIP release.

## Teaser

Inhibition of G_i_-type G proteins expressed by gastrointestinal K cells promotes insulin release and lowers blood glucose levels.

## INTRODUCTION

Glucose-dependent insulinotropic polypeptide (GIP) and glucagon-like peptide-1 (GLP-1) are the key hormones that mediate the so-called incretin effect, which is defined as the amplification of insulin secretion after oral versus intravenous glucose administration (*1*). GIP is the predominant incretin responsible for enhancing insulin secretion in response to a carbohydrate-rich meal in healthy adults (*2*). Both GLP-1 and GIP are secreted from specialized enteroendocrine cells (L and K cells, respectively) and are released into the circulation postprandially (*3*–*5*). GIP, similar to GLP-1, acts on specific G protein–coupled receptors (GPCRs) expressed by pancreatic β cells to enhance insulin secretion and maintain euglycemia (*1*, *4*, *6*, *7*). Elevated plasma GIP levels cause additional beneficial physiological effects including, for example, improved bone health (*8*, *9*) and increased thermogenic activity of brown fat (*10*) and anti-inflammatory effects in mice (*11*).

During the past two decades, incretin receptor agonists have emerged as powerful drugs for the treatment of type 2 diabetes (T2D) and obesity (*12*–*14*). Historically, GLP-1 receptor (GLP-1R) agonists (e.g., liraglutide or semaglutide) have attracted most attention, primarily due to early observations that these drugs exert strong antidiabetic and appetite-suppressing effects (*12*–*14*). In contrast to GLP-1Rs, GIP receptors (GIPRs) received relatively little attention as potential therapeutic targets in the past. One reason was that GIPR agonists, when given alone, showed limited efficacy in individuals with obesity or T2D (*15*) [reviewed in (*4*)]. During the past years, highly efficacious drugs have been developed that target both GIPRs and GLP-1Rs simultaneously. The first member of this class of drugs is tirzepatide, which has been approved by the US Food and Drug Administration for the treatment of T2D and obesity (*16*–*18*). Clinical studies have shown that tirzepatide is more efficacious than semaglutide and other GLP-1R agonists in the approved doses (*16*, *17*, *19*, *20*).

Somewhat unexpectedly, GIPR antagonists can also cause reductions in body weight in obese individuals, besides other beneficial metabolic effects (*21*–*23*), raising the question of how both GIPR agonists and antagonists can have beneficial effects on glucose and energy homeostasis. Recent studies demonstrated that GIPR agonists and antagonists act on different targets in the brain, thus shedding light on the so-called “GIPR agonist/antagonist paradox” (*24*–*26*).

During the past few decades, the development of chemically modified incretin receptor ligands as antidiabetic and antiobesity drugs has been the major focus of many industrial and academic laboratories. Individuals with bariatric surgery display elevated plasma levels of incretins that are predicted to cause major improvements in glucose and energy homeostasis. While the beneficial metabolic roles of elevated plasma GLP-1 levels have been reported in several studies (*27*, *28*), more recent data suggest that enhanced GIP release may also contribute to the beneficial metabolic effects of bariatric surgery (*29*, *30*). Moreover, two recent studies reported that selective stimulation of GIP release from K cells in mice in vivo led to major metabolic improvements in obese and diabetic mice (*31*, *32*). For these reasons, a better understanding of the cellular mechanisms that regulate the release of endogenous GIP in vivo is of considerable translational relevance.

Similar to all other cell types, enteroendocrine K cells express dozens of GPCRs on their cell surface (*7*, *33*, *34*). GPCRs are excellent drug targets, as indicated by the fact that about one third of all drugs used in the clinic target one or more GPCR subtypes (*35*). A specific GPCR exerts its cellular effects via activation of one or more of the four major families of heterotrimeric G proteins (G_q_, G_s_, G_12_, and G_i_) (*36*, *37*). K cells express several members of the G_i_ family of G proteins (G protein α subunits: α_i1_, α_i2_, α_i3_, α_o1_, and α_z_) (fig. S1). Following GPCR-mediated activation, G_i_-type G proteins not only inhibit distinct downstream effector proteins or ion channels (e.g., adenylyl cyclase or certain voltage-dependent calcium channels) but can also stimulate the activity of a subclass of potassium channels [G protein-coupled inwardly rectifying potassium (GIRK) channels] and cause additional cellular effects (*37*). At present, the in vivo metabolic roles of G_i_-type G proteins expressed by enteroendocrine K cells remain unknown.

To address this question, we initially used a chemogenetic approach. Specifically, we generated a mouse model that expressed a G_i_-coupled DREADD (designer receptor exclusively activated by a designer drug) (GiD) (*38*, *39*) selectively in K cells. We also generated a loss-of-function mouse model that lacked functional G_i_-type G proteins in K cells.

Systematic metabolic studies showed that G proteins of the G_i_ family endogenously expressed by K cells play key roles in regulating glucose homeostasis and related metabolic functions. Collectively, our data suggest that agents able to inhibit G_i_ signaling in K cells may prove beneficial to elevate plasma GIP levels for therapeutic purposes.

## RESULTS

### Generation and characterization of K-GiD mice

**Fig. 1.**
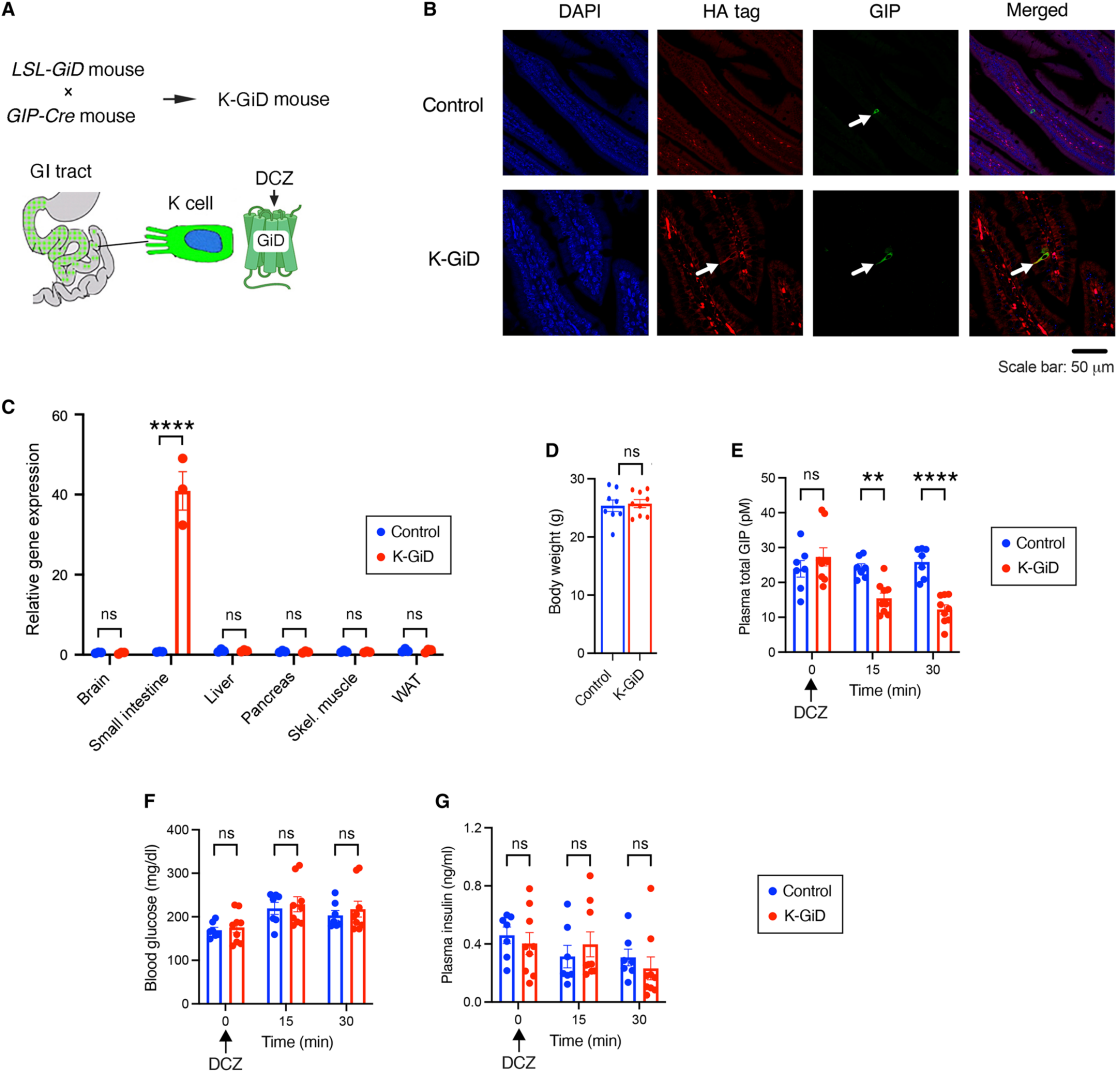
Selective activation of K cell G_i_ signaling decreases GIP secretion in vivo. (**A**) Scheme depicting the generation of mice selectively expressing the GiD DREADD (GiD) in K cells (K-GiD mice). Details regarding mouse genotypes, control mice, etc., are given under Materials and Methods. (**B**) Transcripts encoding GiD are detectable in the small intestine of K-GiD mice but not in other metabolically relevant tissues. Gene expression data were obtained via qRT-PCR and normalized relative to the expression of mouse *36B4* or *Rplp0* (housekeeping genes) (*n* = 3). White arrows point at individual K cells in the endothelial layer. (**C**) Immunohistochemical images of intestinal (duodenal) crypts showing colocalization of GIP and GiD (detected with an anti-HA antibody directed against the HA tag fused to the N terminus of GiD) in the duodenum of K-GiD mice but not control mice. (**D**) Body weight of 8-week-old K-GiD mice and control littermates (males) consuming regular chow. (**E** to **G**) Treatment of K-GiD mice and control littermates with a single oral dose of DCZ (10 μg/kg) after a 6-hour fast. Changes in plasma GIP (E), blood glucose (F), and plasma insulin (G) levels were monitored at the indicated time points. All experiments were carried out with male mice (age, 8 weeks). Data are given as means ± SEM (control mice, *n* = 7; K-GiD mice, *n* = 9). **P < 0.01 and ****P < 0.0001, as compared with the corresponding control group [two-way ANOVA followed by Tukey post hoc analysis (E) to (G) or two-tailed Student’s *t* test (C) and (D), respectively]. ns, no statistically significant difference; WAT, white adipose tissue; GI, gastrointestinal; DAPI, 4′,6-diamidino-2-phenylindole.

To generate transgenic mice that selectively express the GiD in enteroendocrine K cells (K-G_i_ DREADD mice; short name: K-GiD mice), we crossed *Rosa26-LSL-hM4Di* mice (LSL-GiD mice) (*40*) with *Gip-Cre* mice that express Cre recombinase under the transcriptional control of the *Gip* promoter (Fig. 1A) (*41*). LSL-GiD mice that lacked the *Gip-Cre* transgene served as control littermates. We used an anti–N-terminal hemagglutinin (HA) tag antibody to detect the expression of GiD in mouse enteroendocrine cells (*42*). Immunofluorescence staining revealed that GiD expression was localized to GIP-positive K cells (Fig. 1B). We found that about 70% of GIP-positive cells expressed the GiD designer receptor [21 of a total of 29 K cells; note that K cells are very rare (*43*); see Materials and Methods for details]. Quantitative reverse transcription polymerase chain reaction (qRT-PCR) studies showed that transcripts encoding GiD were detectable in the small intestine of K-GiD mice but not in several other metabolically relevant tissues (Fig. 1C).

K-GiD mice and their control littermates did not differ significantly in body weight (diet: regular chow) (Fig. 1D). To investigate whether activation of G_i_ signaling in K cells affected endogenous GIP levels, both groups of mice received an oral dose of deschloroclozapine (DCZ; 10 μg/kg), a selective GiD agonist that is otherwise pharmacologically inert (*44*). Before DCZ administration, K-GiD mice and their control littermates did not differ significantly in plasma GIP, blood glucose, and plasma insulin levels (Fig. 1, E to G). In contrast, DCZ treatment of K-GiD mice, but not of control littermates, resulted in a significant reduction in plasma GIP levels (Fig. 1E). DCZ administration had no significant effect on blood glucose and plasma insulin levels in K-GiD and control mice (Fig. 1, F and G). These data suggest that acute activation of G_i_ signaling in K cells strongly inhibits GIP release.

### Activation of K cell G_i_ signaling results in impaired glucose tolerance in lean K-GiD mice

**Fig. 2.**
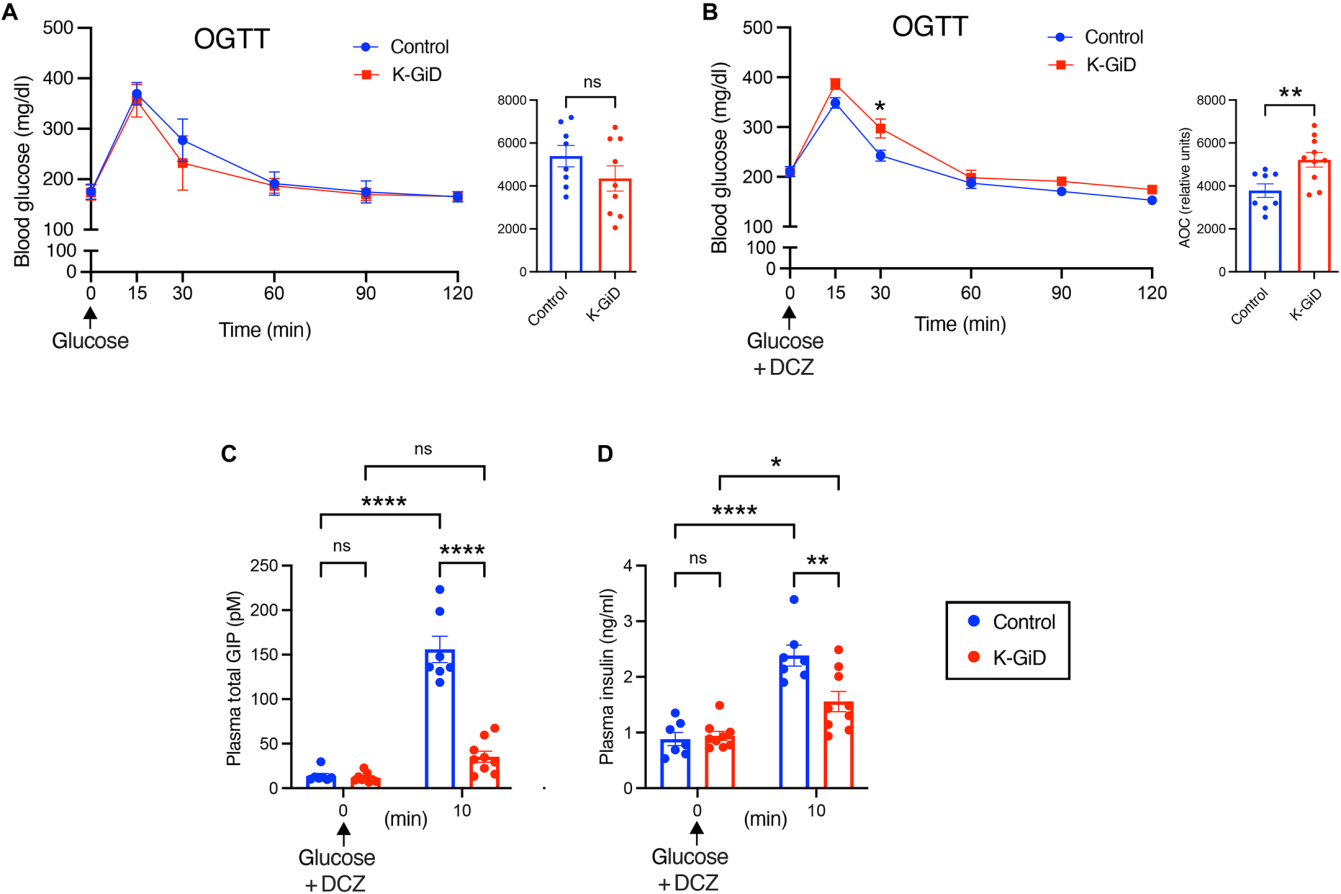
Activation of K cell G_i_ signaling results in impaired glucose tolerance in lean K-GiD mice. (**A** and **B**) Oral glucose tolerance tests (OGTTs). In (A), K-GiD mice and control littermates received an oral glucose bolus (2 g/kg). In (B), both groups of mice were cotreated with oral glucose plus DCZ (10 μg/kg). (**C** and **D**) Plasma hormone levels after cotreatment of K-GiD and control mice with oral glucose (2 g/kg) plus DCZ (10 μg/kg). Plasma GIP (C) and insulin (D) levels were measured at the indicated time points. All experiments were carried out with male mice (age, 9 to 14 weeks) after a 6-hour fast. Data are given as means ± SEM (control mice, *n* = 7; K-GiD mice, *n* = 9). *P < 0.05, **P < 0.01, and ****P < 0.0001, as compared with the corresponding control group {two-way ANOVA followed by Tukey post hoc analysis (A) to (D) or two-tailed Student’s *t* test (A) and (B) [area of the curve (AOC) bars], respectively}.

To explore whether stimulation of K cell G_i_ signaling affected glucose tolerance, we treated K-GiD and control mice with an oral glucose bolus (2 g/kg) either in the absence or presence of DCZ (10 μg/kg) (diet: regular chow). In the absence of DCZ cotreatment, glucose tolerance did not differ significantly between K-GiD and control mice (Fig. 2A). On the other hand, K-GiD mice cotreated with glucose plus DCZ showed a significant impairment in glucose tolerance, when compared to control mice treated in the same fashion (Fig. 2B). Insulin tolerance tests showed that K-GiD and control mice did not differ significantly in peripheral insulin sensitivity [insulin dose, 0.75 U/kg, intraperitoneally (ip)] either in the absence or presence of DCZ (10 μg/kg) (fig. S2, A and B). In control mice, oral coadministration of glucose and DCZ resulted in a very robust elevation in plasma GIP levels (Fig. 2C). In contrast, the same treatment failed to cause a significant increase in plasma GIP levels in K-GiD mice (Fig. 2C). In agreement with this observation, glucose-induced insulin secretion (GSIS) was significantly reduced in K-GiD mice cotreated with glucose plus DCZ, as compared with control mice treated in the same fashion (Fig. 2D).

### Activation of K cell G_i_ signaling under more physiological conditions has detrimental metabolic effects

**Fig. 3.**
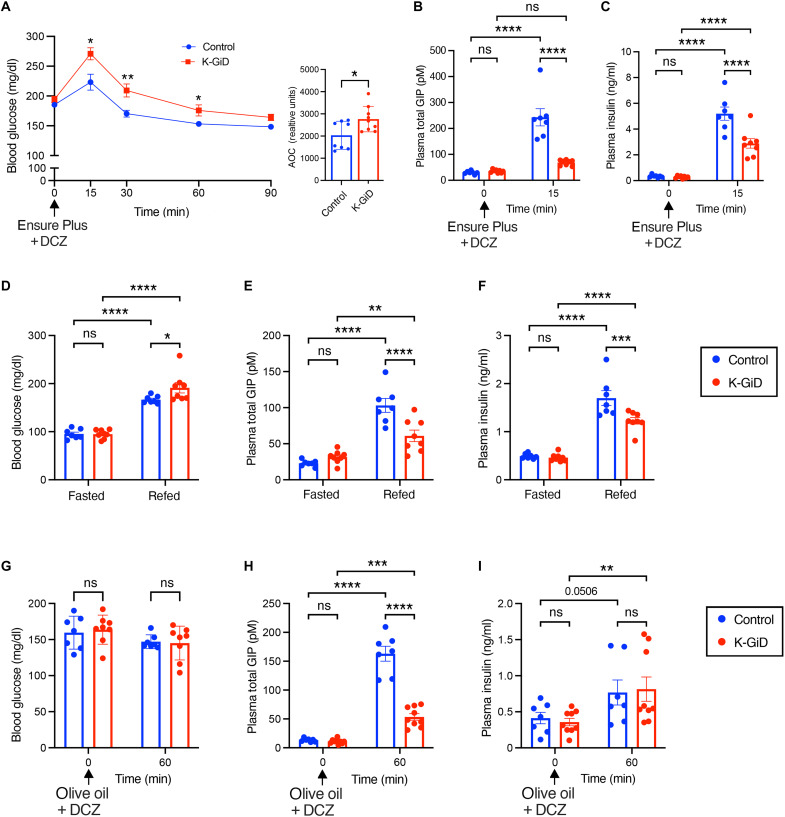
Detrimental metabolic effects of K cell G_i_ signaling in response to a mixed meal, refeeding, or olive oil ingestion. (**A**) Mixed meal (Ensure Plus) tolerance test. After a 6-hour fast, K-GiD mice and control littermates received an oral bolus of Ensure Plus (10 ml/kg) containing DCZ (10 μg/kg), followed by the monitoring of blood glucose levels. (**B** and **C**) Effect of oral cotreatment of K-GiD and control mice with Ensure Plus (10 ml/kg) plus DCZ (10 μg/kg) on plasma GIP (B) and insulin (C) levels. (**D** to **F**) Refeeding studies. Following a 24-hour fast, K-GiD and control mice had free access to regular chow for 2 hours. All mice received oral DCZ (10 μg/kg) before refeeding. Blood glucose (D), plasma GIP (E), and plasma insulin (F) levels were measured at the end of the fasting and refeeding periods. (**G** to **I**) Olive oil–induced changes in blood glucose and plasma hormone levels. After a 6-hour fast, K-GiD and control mice received an oral bolus of olive oil (10 μl/g) supplemented with DCZ (10 μg/kg). Blood glucose (G), plasma GIP (H), and plasma insulin (I) levels were measured 60 min later. All experiments were carried out with male mice (age, 15 to 17 weeks). Data are given as means ± SEM (control mice, *n* = 7; K-GiD mice, *n* = 9). *P < 0.05, **P < 0.01, ***P < 0.01, and ****P < 0.0001, as compared with the corresponding control group [two-way ANOVA followed by Tukey post hoc analysis (A) to (I) or two-tailed Student’s *t* test (A) (AOC data), respectively].

We also treated mice with a mixed liquid meal (Ensure Plus) to study food-induced changes in glucose homeostasis under more physiological conditions. Specifically, K-GiD mice and their control littermates received an oral bolus of Ensure Plus (10 ml/kg) supplemented with DCZ (10 μg/kg), followed by the monitoring of blood glucose levels. Under these experimental conditions, K-GiD mice showed significantly impaired glucose tolerance as compared to their control littermates (Fig. 3A). In control mice, Ensure Plus and DCZ cotreatment resulted in a statistically significant elevation in plasma GIP levels (Fig. 3B). In contrast, the same treatment had no significant effect on plasma GIP levels in K-GiD mice (Fig. 3B). Similarly, the increase in plasma insulin levels caused by the mixed meal (plus DCZ) was significantly smaller in K-GiD mice as compared to their control littermates (Fig. 3C).

In a different set of experiments, we subjected K-GiD mice and their control littermates to a 24-hour fast. At the end of this period, mice received oral DCZ (10 μg/kg) 30 min before they were given free access to regular chow for a 2-hour period. Under these experimental conditions, K-GiD mice displayed significantly higher blood glucose at the end of the refeeding period, as compared to control littermates (Fig. 3D). At the same time, the refeeding-induced increases in plasma GIP and insulin levels were significantly less pronounced in K-GiD mice (Fig. 3, E and F). Together, the findings indicate that activation of K cell G_i_ signaling under more physiological conditions (consumption of a mixed meal or refeeding after a prolonged fast) has detrimental effects on GIP and insulin release, leading to impaired glucose homeostasis.

### Olive oil–induced increases in plasma GIP levels are greatly reduced during K cell G_i_ signaling

It is well known that long-chain fatty acids promote GIP release via activation of GPCRs expressed on the surface of K cells (*4*, *45*). These receptors include free fatty acid receptor 1 (FFAR1) and G protein-coupled receptor 119 (GPR119), which are linked to G_q/11_ and G_s_, respectively (*46*). To explore the effect of K cell G_i_ signaling on fatty acid–induced GIP release, we treated K-GiD mice and their control littermates with olive oil (10 ml/kg) supplemented with DCZ (10 μg/kg), followed by the monitoring of blood glucose and plasma hormone levels 60 min later. Under these experimental conditions, DCZ-induced activation of K cell G_i_ signaling had little or no effect on blood glucose and plasma insulin levels (Fig. 3, G and I). In contrast, the olive oil–induced increase in plasma GIP levels was greatly reduced in DCZ-treated K-GiD mice as compared to control mice treated in the same fashion (Fig. 3H). The observation that the low GIP levels displayed by K-GiD mice did not affect plasma insulin and blood glucose levels can be explained by the fact that GIP-induced increases in insulin secretion require elevated blood glucose levels (*4*).

### Metabolic effects of chronic activation of K cell G_i_ signaling

We next investigated the metabolic effects of chronic activation of K cell G_i_ signaling. Specifically, K-GiD mice and their control littermates consumed drinking water containing DCZ (10 mg/liter) for 2 weeks (diet: regular chow). At the end of the DCZ treatment period, body weight and acute food intake did not differ significantly between the two groups of mice (fig. S3, A and B). However, chronic activation of K cell G_i_ signaling resulted in significantly decreased plasma GIP levels as compared to the corresponding control mice (fig. S3D). In contrast, blood glucose and plasma insulin levels remained unaffected under these experimental conditions (fig. S3, C and E).

At the end of the 2-week DCZ treatment period, we also measured oral glucose tolerance in the two groups of mice. We found that glucose tolerance was significantly impaired in K-GiD mice as compared to control littermates (fig. S3F). In insulin tolerance tests, the two groups of mice showed similar peripheral insulin sensitivity after chronic DCZ consumption (fig. S3G). After an oral glucose bolus (2 g/kg), K-GiD mice that had been maintained on DCZ drinking water for 2 weeks showed significantly elevated blood glucose levels, associated with significantly reduced plasma GIP and insulin levels, as compared to control mice treated in the same manner (fig. S3, H to J). These data indicate that chronic activation of K cell G_i_ signaling mimics the detrimental metabolic effects observed after acute stimulation of this pathway.

### Acute activation of K cell G_i_ signaling impairs glucose tolerance in obese mice

**Fig. 4.**
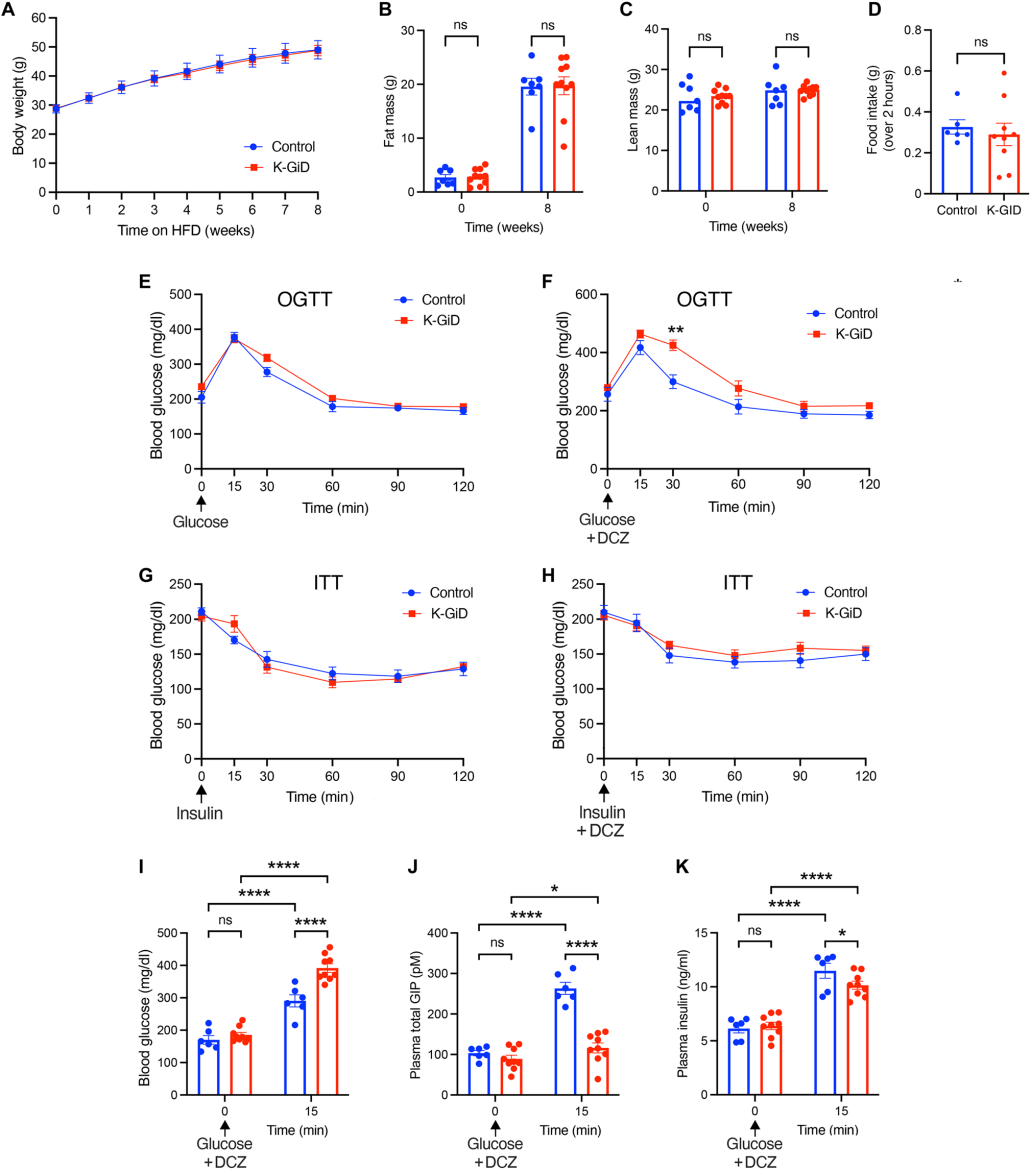
Acute activation of K cell G_i_ signaling further impairs glucose tolerance in obese K-GiD mice. (**A**) Body weight gain of K-GiD and control mice (males) when maintained on an HFD. (**B** and **C**) Fat mass (B) and lean mass (C) before and 8 weeks after HFD feeding. (**D**) Food intake studies. Following a 24-hour fast, HFD K-GiD and control mice had free access to regular chow (this diet has a higher carbohydrate content than HFD) for 2 hours. (**E** and **F**) OGTT studies. In (E), HFD K-GiD mice and control littermates received an oral glucose bolus (1 g/kg). In (F), both groups of mice were cotreated with oral glucose plus DCZ (10 μg/kg). (**G** and **H**) ITT studies. In (G), HFD K-GiD mice and control littermates received a single intraperitoneal injection of insulin (1.5 U/kg). In (H), both groups of mice were coinjected intraperitoneally with insulin (1.5 U/kg) plus DCZ (10 μg/kg). (**I** to **K**) Blood glucose and plasma hormone measurements. HFD K-GiD and control mice received oral glucose (1 g/kg) supplemented with DCZ (10 μg/kg). Blood glucose (I), plasma GIP (J), and plasma insulin (K) levels were measured 15 min later. All experiments were carried out with male mice (age, 16 to 22 weeks) after a 6-hour fast except for ITT studies (4-hour fast). Data are given as means ± SEM (control mice, *n* = 6; K-GiD mice, *n* = 9). *P < 0.05, **P < 0.01, and ****P < 0.0001, as compared with the corresponding control group [two-way ANOVA followed by Tukey post hoc analysis (A) to (C) and (E) to (K) or two-tailed Student’s *t* test (D), respectively].

We next carried out a series of metabolic studies with K-GiD mice and control littermates that had been consuming a high-fat diet (HFD) for 8 weeks, resulting in obesity and impaired glucose homeostasis. At the end of the HFD feeding period, K-GiD and control mice did not differ significantly in body weight, body composition, and food intake (Fig. 4, A to D). When treated with an oral bolus of glucose alone (1 g/kg), the two groups of mice showed very similar glucose excursions (Fig. 4E). In contrast, after oral coadministration of glucose (1 g/kg) with DCZ (10 μg/kg), the K-GiD mice showed a statistically significant impairment in glucose tolerance as compared to control littermates (Fig. 4F). K-GiD mice and control littermates showed similar decreases in blood glucose levels in insulin tolerance tests, independent of whether the mice received insulin alone or in combination with DCZ (10 μg/kg) (Fig. 4, G and H). Oral cotreatment of glucose and DCZ resulted in a statistically significant increase in blood glucose levels in K-GiD mice, associated with significantly reduced increases in plasma GIP and insulin levels, as compared to control littermates (Fig. 4, I to K).

### Acute activation of K cell G_i_ signaling in obese mice results in several other metabolic deficits

We next exposed obese K-GiD mice and control littermates to a series of additional, more physiological interventions known to increase plasma GIP levels. Initially, both groups of mice that had been maintained on an HFD for 14 weeks received a mixed meal (Ensure Plus; 10 ml/kg orally) supplemented with DCZ (10 μg/kg). In this experimental setup, K-GiD mice showed a statistically significant increase in blood glucose excursions as compared to control mice (fig. S4A). In addition, obese K-GiD mice cotreated with Ensure Plus and DCZ displayed significantly reduced increases in plasma GIP and insulin levels as compared to control littermates (fig. S4, B and C). We next fasted both groups of mice for 24 hours, followed by an oral DCZ bolus (10 μg/kg) and a 2-hour refeeding period (diet: regular chow). Following refeeding, K-GiD mice exhibited significantly higher blood glucose levels as compared to control littermates (fig. S4D). K-GiD mice also showed reduced increases in plasma GIP and insulin levels, although the effect on insulin failed to reach statistical significance (fig. S4, E and F).

To investigate whether acute activation of K cell G_i_ signaling interfered with fatty acid–induced GIP release in obese mice, HFD K-GiD and control mice received an oral bolus of olive oil (10 ml/kg) supplemented with DCZ (10 μg/kg). Blood glucose and plasma hormone levels were determined 60 min later. Under these experimental conditions, the two groups of mice showed similar blood glucose and plasma insulin levels (fig. S4, G and I). However, K-GiD mice displayed significantly reduced plasma GIP levels as compared to control mice (fig. S4H). These data indicate that activation of K cell G_i_ signaling in obese mice results in similar metabolic deficits as observed with lean mice.

### Generation and initial characterization of mutant mice deficient in K cell G_i_ signaling

**Fig. 5.**
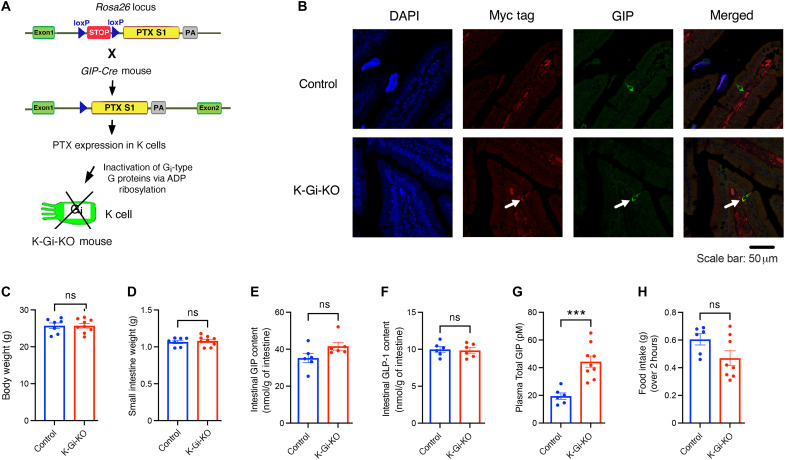
Inactivation of K cell G_i_ signaling results in increased plasma GIP levels. (**A**) Scheme depicting the generation of mice selectively expressing the S1 catalytic subunit of PTX (S1 PTX) in K cells (K-Gi-KO mice). Details regarding mouse genotypes, control mice, etc., are given under Materials and Methods. (**B**) Immunohistochemical images of intestinal (duodenal) crypts showing colocalization of GIP and S1 PTX (detected with an anti-myc antibody directed against the myc epitope tag fused to the C terminus of S1 PTX) in the duodenum of K-Gi-KO mice but not control mice. White arrows point at individual K cells in the endothelial layer. (**C** to **G**) Characterization of K-Gi-KO mice and control littermates (8-week-old males; diet: regular chow). Body weight (C), small intestine weight (D), intestinal GIP (E) and GLP-1 content (F), and plasma GIP (G) levels. (**H**) Acute food intake studies. Following a 24-hour fast, K-Gi-KO and control mice had free access to food for 2 hours (8-week-old males; diet: regular chow). Mice used for measuring intestinal GIP and GLP-1 content were 16 weeks old. Data are given as means ± SEM (control mice, *n* = 6; K-Gi-KO mice, *n* = 9). ***P < 0.01, as compared with the corresponding control group (two-tailed Student’s *t* test). PA, polyadenylation site.

To further explore the physiological role of K cell G_i_ signaling, we generated mice in which the α subunits of G proteins of the G_i_ family had been selectively inactivated in K cells. To generate this mouse strain, we expressed the S1 subunit of pertussis toxin (PTX) selectively in K cells by introducing the *Gip-Cre* transgene (*41*) into the genome of *ROSA26^PTX^* mice (Fig. 5A) (*42*). In the following, we refer to these mice simply as “K-Gi-KO mice.” *ROSA26^PTX^* littermates that did not harbor the *Gip-Cre* transgene served as control animals for all experiments.

Since the S1 subunit of PTX was engineered to contain a C-terminal myc epitope tag (*42*), we used an anti-myc antibody to detect PTX in mouse enteroendocrine cells. Immunofluorescence staining revealed that PTX expression was selectively localized to GIP-positive K cells (Fig. 5B). We found that about 70% of GIP-positive cells expressed the PTX protein (22 of a total of 30 K cells; note that K cells are very rare; see Materials and Methods for details). Demonstrating the inhibitory effect of PTX on G_i_-mediated signaling in isolated K cells is technically very challenging. However, previous studies have shown that expression of this protein in various cell types prevents receptor-mediated activation of G_i_-type G proteins (*42*, *47*, *48*).

K-Gi-KO mice and control littermates did not differ significantly in body weight and small intestinal weight (Fig. 5, C and D). Moreover, the two groups of mice showed similar intestinal GIP and GLP-1 content (Fig. 5, E and F). However, plasma GIP levels were ∼2-fold higher in K-Gi-KO than in control mice (Fig. 5G). Food intake (diet: regular chow) did not differ significantly between the two groups of mice (Fig. 5H).

### Loss of K cell G_i_ signaling improves glucose tolerance in lean mice

**Fig. 6.**
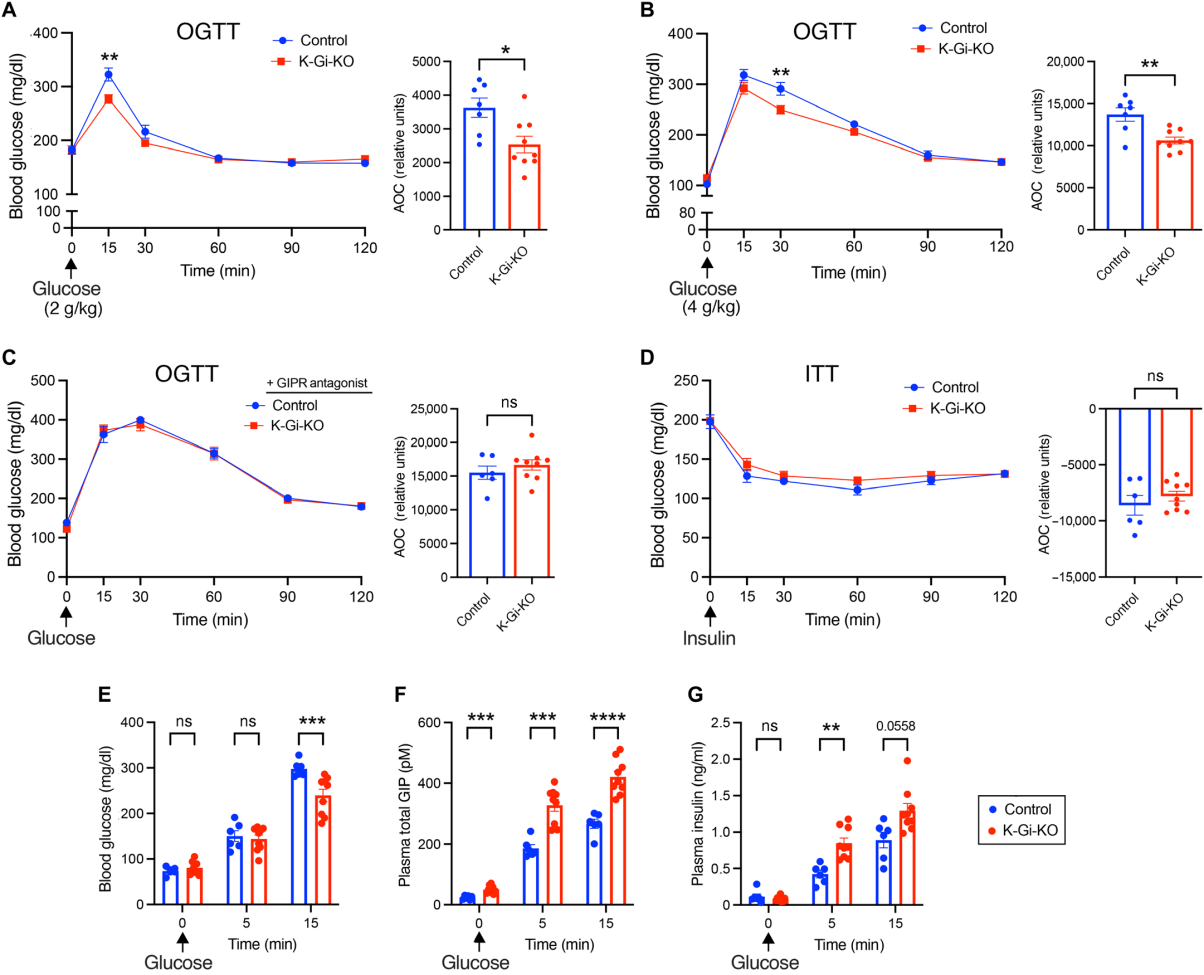
Inhibition of K cell G_i_ signaling results in improved glucose tolerance and elevated plasma GIP and insulin levels. (**A** to **C**) OGTT studies. In (A) and (B), K-Gi-KO mice and control littermates received an oral glucose bolus (2 and 4 g/kg, respectively). In (C), both groups of mice were first injected with a GIPR antagonist (GIPA-2; 1500 nmol/kg, ip). Thirty minutes later, all mice received an oral glucose bolus (2 g/kg). (**D**) ITT. K-Gi-KO mice and control littermates were treated with insulin (0.75 U/kg, ip). (**E** to **G**) Measurement of blood glucose and plasma hormone levels. Following treatment of K-Gi-KO and control mice with oral glucose (2 g/kg), blood glucose (E), plasma GIP (F), and plasma insulin (G) levels were measured at the indicated time points. All experiments were carried out with male mice (age, 9 to 13 weeks) after a 6-hour fast except for ITT studies (4-hour fast). Data are given as means ± SEM (control mice, *n* = 7; K-Gi-KO mice, *n* = 9). *P < 0.05, **P < 0.01, ***P < 0.01, and ****P < 0.0001, as compared with the corresponding control group [two-way ANOVA followed by Tukey post hoc analysis (A) to (G) or two-tailed Student’s *t* test (A) to (D) (OC data), respectively].

To determine whether the lack of K cell G_i_ signaling affected glucose tolerance in lean mice, we treated K-Gi-KO mice and control littermates with two different doses of glucose (2 and 4 g/kg orally). Independent of the glucose dose administered, K-Gi-KO mice showed significantly improved glucose tolerance as compared to control littermates (Fig. 6, A and B). To test whether this beneficial metabolic effect was caused by elevated plasma GIP levels, we treated both groups of mice with a selective GIPR antagonist (GIPA-2; 1500 nmol/kg, ip) (*49*). Thirty minutes later, all mice received an oral bolus of glucose (4 g/kg), followed by the monitoring of blood glucose levels [oral glucose tolerance test (OGTT)]. As shown in Fig. 6C, pretreatment with the GIPR antagonist abolished the improvement in glucose tolerance displayed by K-Gi-KO mice, indicating that this phenotype was most likely due to elevated plasma GIP levels. K-Gi-KO mice and control littermates showed similar peripheral insulin sensitivity (ITT) (Fig. 6D).

We also measured blood glucose and plasma hormone levels 5 and 15 min after administration of oral glucose (4 g/kg). At the 15-min time point, the increase in blood glucose levels was significantly smaller in K-Gi-KO mice than in control littermates (Fig. 6E). Moreover, plasma GIP and insulin levels were elevated in K-Gi-KO mice at both time points (this effect failed to reach statistical significance for plasma insulin at 15 min) (Fig. 6, F and G). These data indicate that inhibition of K cell G_i_ signaling triggers enhanced GIP release which stimulates GSIS, resulting in improved glucose homeostasis.

### Deficient K cell G_i_ signaling improves glucose homeostasis under more physiological conditions

**Fig. 7.**
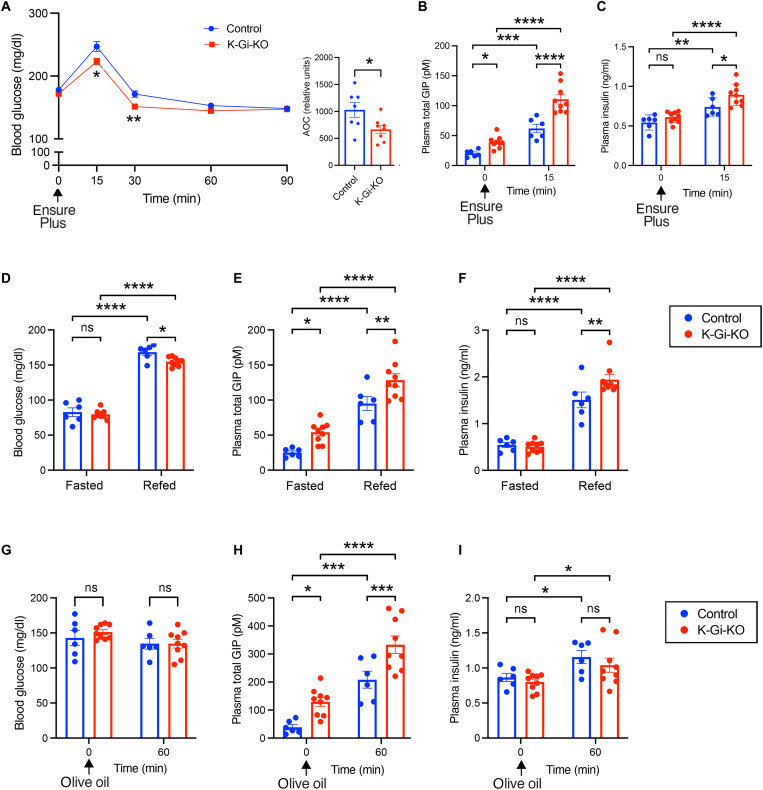
Loss of K cell G_i_ signaling in lean mice causes metabolic improvements in response to a mixed meal, refeeding, or olive oil ingestion. (**A**) Mixed meal (Ensure Plus) tolerance test. K-Gi-KO mice and control mice littermates received an oral bolus of Ensure Plus (10 ml/kg), followed by the measurement of blood glucose levels. (**B** and **C**) Plasma hormone measurements. Mixed meal-induced changes in plasma GIP (B) and insulin (C) levels were measured at the indicated time points. (**D** to **F**) Refeeding studies. After a 24-hour fast, K-Gi-KO and control mice had free access to regular chow for 2 hours. Blood glucose (D), plasma GIP (E), and plasma insulin (F) levels were determined immediately before and after the refeeding period. (**G** to **I**) Olive oil–induced changes in blood glucose and plasma hormone levels. K-Gi-KO and control mice littermates received an oral bolus of olive oil (10 μl/g). Blood glucose (G), plasma GIP (H), and plasma insulin (I) levels were measured at the indicated time points. Unless noted otherwise, all experiments were carried out with male mice (age, 14 to 16 weeks) after a 6-hour fast. Data are given as means ± SEM (control mice, *n* = 7; K-Gi-KO mice, *n* = 9). *P < 0.05, **P < 0.01, ***P < 0.01, and ****P < 0.0001, as compared with the corresponding control group [two-way ANOVA followed by Tukey post hoc analysis (A) to (I) or two-tailed Student’s *t* test (A) (AOC data), respectively].

To investigate whether the loss of K cell G_i_ signaling affected glycemia in response to a mixed meal, K-Gi-KO mice and control littermates received an oral bolus of Ensure Plus (10 ml/kg). In this experiment, K-Gi-KO mice showed significantly improved glucose tolerance as compared to control littermates (Fig. 7A). Ingestion of the mixed meal also led to significantly greater increases in plasma GIP and insulin levels in K-Gi-KO mice (Fig. 7, B and C).

We also subjected K-Gi-KO and control mice to a refeeding test. Specifically, both groups of mice were fasted for 24 hours and then allowed access to food (diet: regular chow) for 2 hours. At the end of the refeeding period, K-Gi-KO mice showed significantly lower blood glucose levels and elevated plasma GIP and insulin levels as compared to control littermates (Fig. 7, D to F). We noted that basal GIP levels were also significantly elevated in fasted K-Gi-KO mice (Fig. 7E).

To study fatty acid–induced GIP release, we treated K-Gi-KO mice and their control littermates with olive oil (oral administration; 10 ml/kg), followed by the monitoring of blood glucose and plasma hormone levels 60 min later. Under these experimental conditions, blood glucose and plasma insulin levels showed no or only minor changes in both groups (Fig. 7, G and I). In contrast, plasma GIP levels were significantly increased both before and after olive oil treatment in K-Gi-KO mice as compared to control mice (Fig. 7H).

### Loss of K cell G_i_ signaling has beneficial metabolic effects in obese mice

**Fig. 8.**
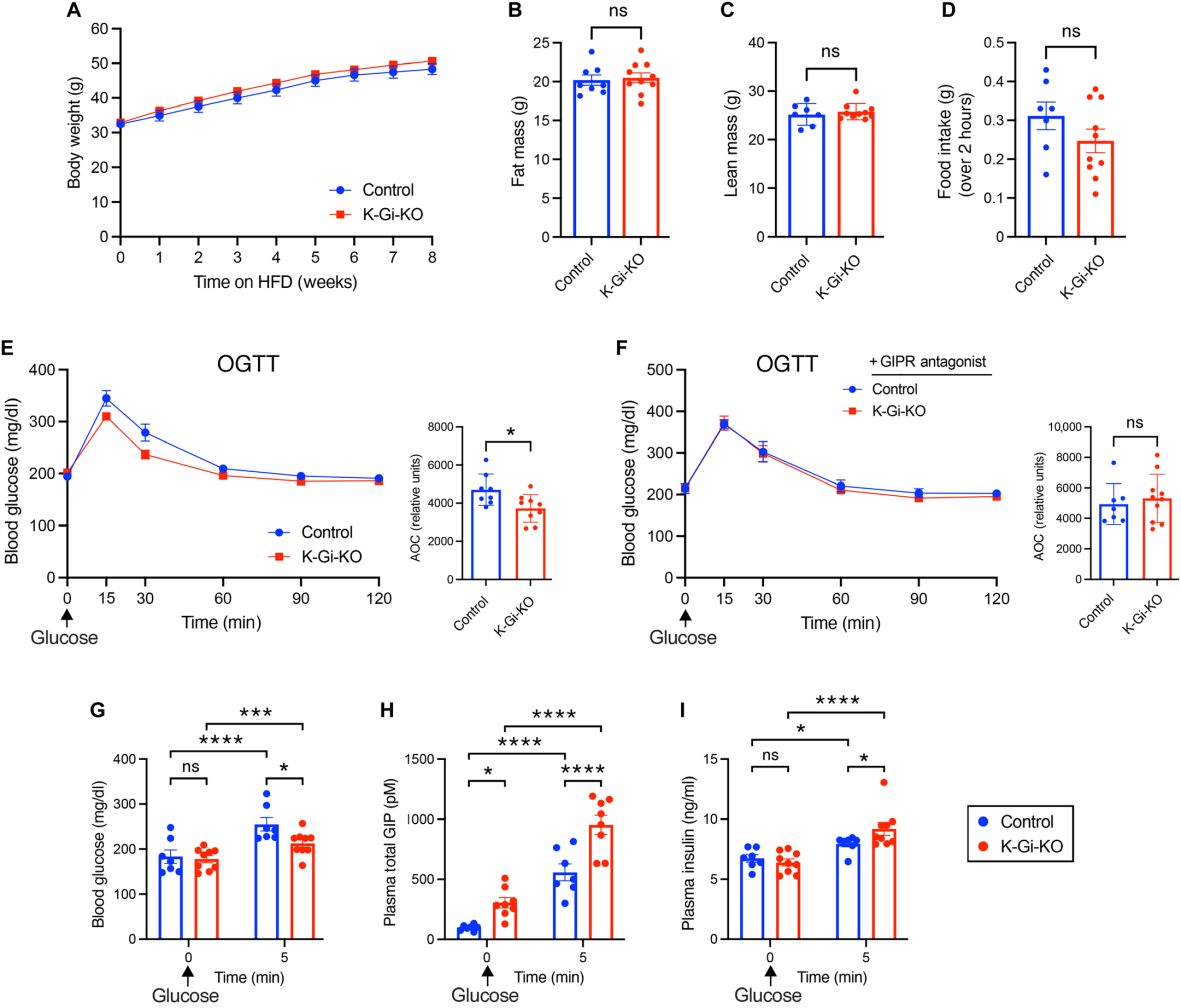
Lack of K cell G_i_ signaling improves glucose tolerance in obese mice. (**A**) Body weight of K-Gi-KO mice and control littermates consuming an HFD for 8 weeks. (**B** and **C**) Fat mass (B) and lean mass (C) after 8 weeks of HFD feeding. (**D**) Acute food intake. Following a 24-hour fast, K-Gi-KO and control mice had free access to regular chow for 2 hours (mice had been maintained on the HFD for 8 weeks). (**E** and **F**) OGTT studies. In (E), both groups of HFD mice received an oral glucose bolus (1 g/kg). In (F), both cohorts of mice were first injected with a GIPR antagonist (GIPA-2; 1500 nmol/kg, ip). Thirty minutes later, all mice received an oral glucose bolus (1 g/kg). (**G** to **I**) Blood glucose and plasma hormone measurements. HFD K-Gi-KO mice and control littermates received an oral glucose bolus (1 g/kg). Blood glucose (G), plasma GIP (H), and plasma insulin (I) levels were measured at the indicated time points. All experiments were carried out with male mice (age, 16 to 19 weeks) after a 6-hour fast except for ITT studies (4-hour fast). Data are given as means ± SEM (control mice, *n* = 8; K-Gi-KO mice, *n* = 9). *P < 0.05, ***P < 0.01, and ****P < 0.0001, as compared with the corresponding control group [two-way ANOVA followed by Tukey post hoc analysis (A) and (E) to (I) or Student’s *t* test (B) to (F), respectively].

To test whether loss of K cell G_i_ signaling improved obesity-induced metabolic impairments, we placed 9-week-old K-Gi-KO mice and control littermates on an HFD for 8 weeks, followed by a series of metabolic tests. Body weight, body composition, and food intake did not differ significantly between the two groups of mice after the HFD feeding period (Fig. 8, A to D). Obese K-Gi-KO mice exhibited significantly improved oral glucose tolerance (glucose dose, 1 g/kg) as compared to their control littermates (Fig. 8E). This beneficial metabolic effect displayed by K-Gi-KO mice was abolished after pretreatment of mice with a selective GIPR antagonist (GIPA-2; 1500 nmol/kg, ip) (Fig. 8F), indicating that the improved glucose tolerance displayed by obese K-Gi-KO mice was dependent on the activation of GIPRs. In agreement with this notion, oral glucose treatment (1 g/kg) not only blunted increases in blood glucose levels (Fig. 8G) but also caused significant further increases in plasma GIP and insulin levels in obese K-Gi-KO mice as compared to obese control mice (Fig. 8, H and I). These data indicate that inhibition of K cell G_i_ signaling improves glucose homeostasis in obese mice in a GIP-dependent fashion.

### Impaired K cell G_i_ signaling in obese results in beneficial metabolic effects under more physiological conditions

**Fig. 9.**
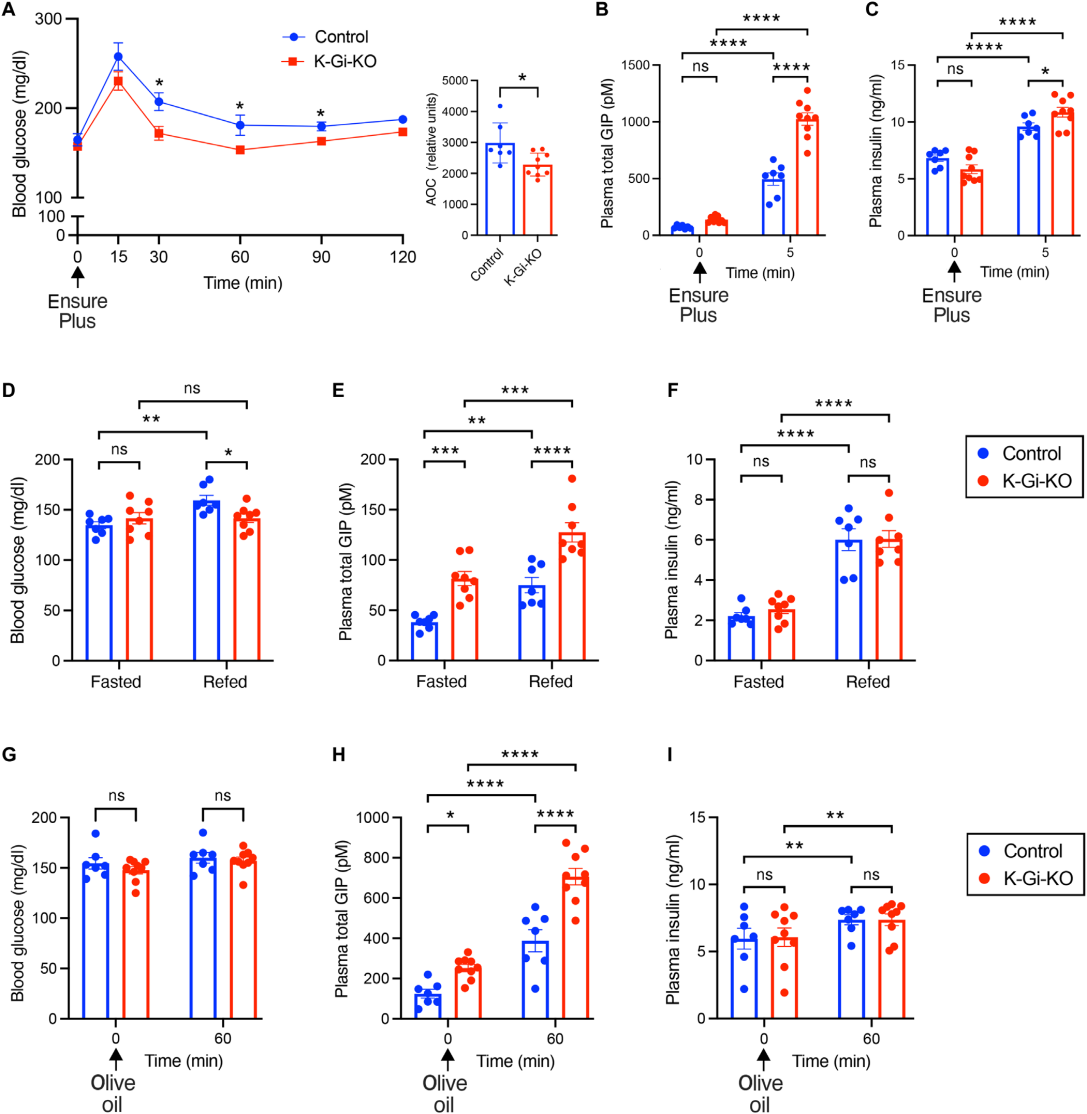
Disruption of K cell G_i_ signaling leads to multiple metabolic improvements in obese mice. K-Gi-KO mice and control littermates were maintained on an HFD for about 12 weeks. (**A**) Mixed meal tolerance test. HFD K-Gi-KO mice and control littermates received an oral bolus of Ensure Plus (10 ml/kg), followed by the monitoring of blood glucose levels. (**B** and **C**) Changes in plasma hormone levels after a mixed meal. HFD K-Gi-KO and control mice received Ensure Plus orally (10 ml/kg). Plasma GIP (B) and insulin (C) levels were measured at the indicated time points. (**D** to **F**) Refeeding studies**.** Following a 24-hour fast, HFD K-GiD and control mice had free access to regular chow (higher carbohydrate content than HFD) for 2 hours. Blood glucose (D), plasma GIP (E), and plasma insulin (F) levels were determined immediately before and after the refeeding period. (**G** to **I**) Olive oil–induced changes in blood glucose and plasma hormone levels. K-Gi-KO mice and control mice littermates received an oral bolus of olive oil (10 μl/g). Blood glucose (G), plasma GIP (H), and plasma insulin (I) levels were measured at the indicated time points. Unless indicated otherwise, all experiments were carried out with male mice (age, 21 to 23 weeks) after a 6-hour fast. Data are given as means ± SEM (*n* = 7 or 8 mice per group). *P < 0.05, **P < 0.01, ***P < 0.001, and ****P < 0.0001, as compared with the corresponding control group [two-way ANOVA followed by Tukey post hoc analysis (A) to (I) or Student’s *t* test (A) (AOC data), respectively].

We next treated obese K-Gi-KO mice and control littermates with a mixed meal (oral bolus of Ensure Plus; 10 ml/kg). We found that K-Gi-KO mice displayed significantly reduced blood glucose excursions as compared to the corresponding control mice (Fig. 9A). Under these experimental conditions, obese K-Gi-KO mice also showed significantly enhanced plasma GIP and insulin levels (Fig. 9, B and C).

We also subjected obese K-Gi-KO and control mice to a refeeding experiment. Following a 24-hour fast, mice were allowed free access to regular chow for 2 hours. At the end of the refeeding period, obese K-Gi-KO mice showed reduced blood glucose but increased plasma GIP levels as compared to obese control littermates (Fig. 9, D and E). Plasma insulin levels did not differ significantly between the two groups of mice (Fig. 9F). The fact that blood glucose levels remained relatively low after refeeding most likely explains why the increase in plasma GIP displayed by the obese K-Gi-KO mice did not trigger further enhanced insulin release compared to the control mice.

To explore whether fatty acid–stimulated GIP release was altered by the lack of K cell G_i_ signaling, we treated obese K-Gi-KO and control littermates with an oral bolus of olive oil (10 ml/kg) and determined blood glucose and plasma hormone levels 60 min later. Under these experimental conditions, blood glucose and plasma insulin levels did not differ significantly between the two groups of mice (Fig. 9, G and I). In contrast, K-Gi-KO mice showed a ∼2-fold increase in plasma GIP levels 60 min after olive oil administration (Fig. 9H), indicating that fatty acid–induced GIP release can be enhanced by inhibiting K cell G_i_ signaling.

### Loss of K cell G_i_ signaling improves glucose tolerance in a mouse model of severe diabetes

We next investigated whether impaired K cell G_i_ signaling could ameliorate hyperglycemia in a mouse model of diabetes. Specifically, we treated 8-week-old male K-Gi-KO mice and control littermates with a low dose of streptozotocin (STZ; 50 mg/kg, ip) or vehicle for 5 consecutive days. This STZ dosing scheme is known to reduce β cell mass by ∼80%, resulting in severe hyperglycemia (*50*, *51*). One week after the initiation of STZ treatment, STZ-treated K-Gi-KO mice showed a significantly smaller increase in blood glucose levels as compared to STZ-treated control mice (fig. S5A). However, at later time points, STZ-treated K-Gi-KO and STZ-treated control mice displayed similar elevations in blood glucose levels (fig. S5A). Pancreatic insulin and glucagon content measured at the conclusion of the experiment showed no significant differences between the two vehicle-treated groups. In contrast, pancreatic insulin content was significantly elevated in STZ-treated K-Gi-KO mice (fig. S5B). On the other hand, pancreatic glucagon content was significantly reduced in this group of mice as compared to STZ-treated control littermates (fig. S5C).

Four weeks after the initiation of STZ/vehicle treatment, we determined blood glucose and plasma hormone levels in all four groups of mice. Consistent with the data shown in fig. S5A, the two STZ-treated groups displayed a similar degree of hyperglycemia (fig. S5D). The vehicle- and STZ-treated K-Gi-KO mice displayed significantly elevated plasma GIP levels as compared to their corresponding control littermates (fig. S5E). However, STZ treatment led to comparable decreases in plasma insulin levels in K-Gi-KO and control mice (fig. S5F). While vehicle-treated K-Gi-KO and control mice showed similar plasma glucagon levels, the STZ-induced increase in plasma glucagon levels was less pronounced in STZ-treated K-Gi-KO mice than in STZ-treated control littermates (fig. S5G). The cellular and molecular mechanisms underlying this phenotype remain to be elucidated.

To investigate whether deficient K cell G_i_ signaling affected glucose tolerance in the STZ diabetes model, we treated all four groups of mice with an oral bolus of glucose (2 g/kg) following an overnight fast. We found that STZ-treated K-Gi-KO mice showed significantly improved glucose tolerance at the 15- and 60-min time points as compared to STZ-treated control littermates (fig. S5H). Moreover, plasma GIP levels were increased by ∼2-fold 10 min after treatment with oral glucose in both vehicle- and STZ-treated K-Gi-KO mice as compared to the corresponding control littermates (fig. S5I). The glucose-induced elevation in plasma insulin levels was significantly increased in vehicle-treated K-Gi-KO mice as compared to vehicle-treated control mice (fig. S5J). Plasma insulin levels also showed a trend (*P* = 0.12) to be elevated in STZ-treated K-Gi-KO mice (fig. S5J). Together, these data indicate that inhibition of K cell G_i_ signaling can lead to a modest improvement in glucose tolerance in a mouse model of severe T2D.

### Glucose-stimulated GIP secretion requires the activation of exchange protein directly activated by 3ʹ,5ʹ-cyclic adenosine monophosphate 2

**Fig. 10.**
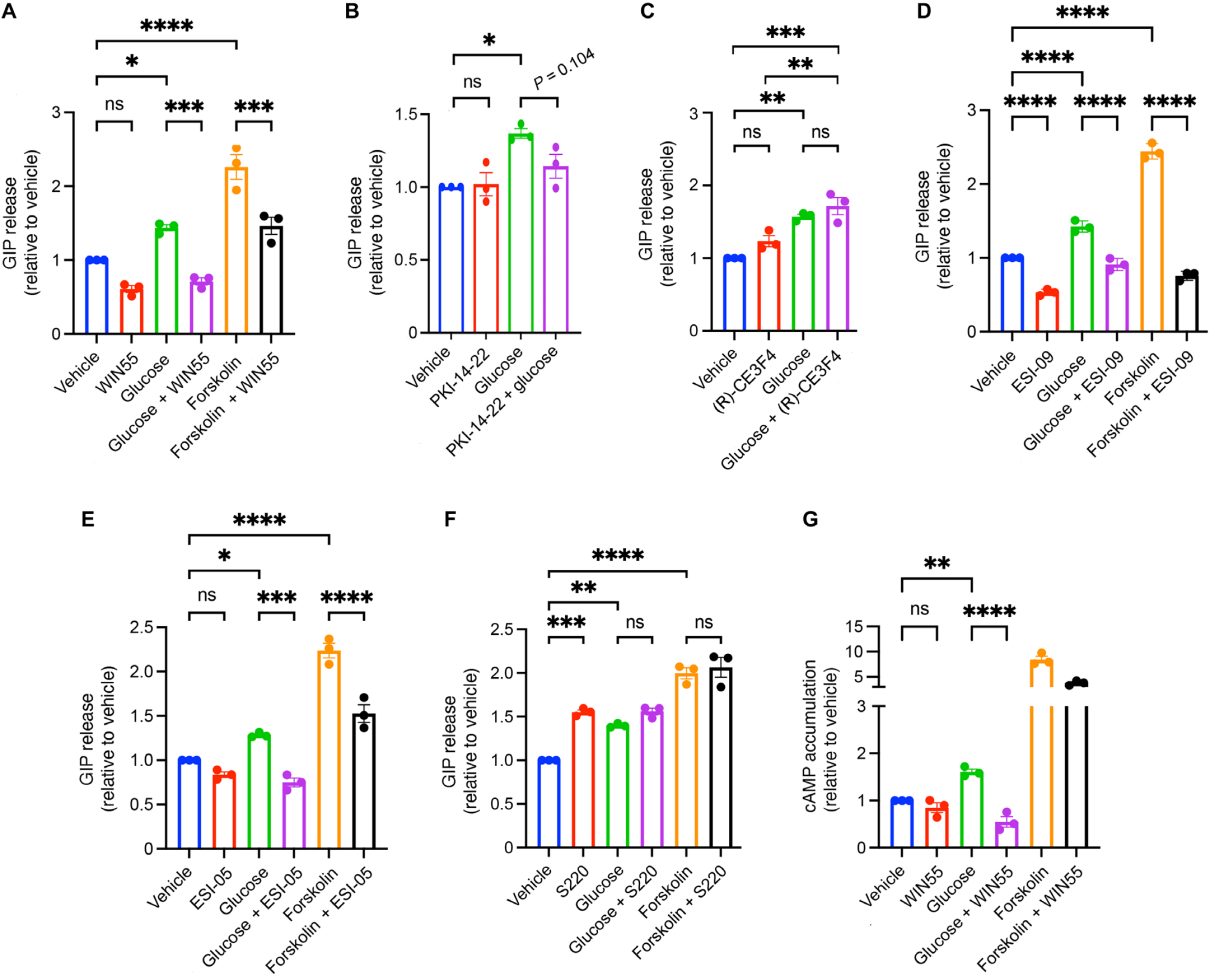
Glucose-stimulated GIP release requires EPAC2 signaling in STC-1 cells. (**A**) Glucose (20 mM)–induced GIP release from STC-1 cells is blocked by a selective cannabinoid receptor agonist (WIN55; 10 μM). (**B** and **C**) Glucose-dependent GIP release from STC-1 cells in the presence of a selective PKA inhibitor (PKI-14-22; 100 nM) (B) or a selective EPAC1 blocker [(R)-CE3F4; 10 μM] (C). (**D** and **E**) Glucose-induced GIP release from STC-1 cells is blocked by selective inhibitors of EPAC1/2 (ESI-09; 10 μM) (D) and EPAC2 (ESI-05; 10 μM) (E). (**F**) A selective EPAC2 activator (S220; 10 μM) mimics the ability of glucose (20 mM) to stimulate GIP secretion from STC-1 cells. Forskolin-stimulated GIP release served as an internal control in most experiments. (**G**) Glucose (20 mM) stimulation of cAMP accumulation in STC-1 cells is abolished in the presence of a selective cannabinoid receptor agonist (WIN55; 10 μM). Data are given as means ± SEM from three independent experiments. *P < 0.05, **P < 0.01, ***P < 0.01, and ****P < 0.0001 (one-way ANOVA followed by Tukey post hoc analysis) (A) to (G), as compared with the corresponding control group.

To explore the molecular mechanisms through which activation of G_i_ signaling affects glucose-induced GIP release, we used the mouse STC-1 enteroendocrine cell line as an in vitro model system (*52*). Besides other G_i_-coupled receptors, the cannabinoid-1 receptor (CB1 receptor) is expressed at high levels in mouse K cells (*53*–*55*). We found that incubation of STC-1 cells with WIN55,212-2 mesylate (WIN55; 10 μM), a selective cannabinoid receptor agonist, completely abolished the glucose-induced increase in GIP secretion (Fig. 10A). In a similar fashion, WIN55 treatment greatly reduced the forskolin (10 μM)–induced increase in GIP release from STC-1 cells (Fig. 10A).

We next examined whether free βγ subunits released after stimulation of G proteins of the G_i_ family contributed to the inhibitory effect of WIN55 on glucose-induced GIP secretion. To address this issue, we treated STC-1 cells with gallein (10 μM), a selective inhibitor of βγ-mediated signaling (*56*). We found that the inhibitory WIN55 response was not affected by treatment with gallein, suggesting that WIN55 suppresses glucose-induced GIP secretion via activated Gα_i_ subunits (fig. S6).

Activation of G_i_-coupled receptors leads to decreased intracellular adenosine 3′,5′-monophosphate (cAMP) levels via inhibition of adenylyl cyclase (*36*). cAMP exerts most of its cellular functions by activating protein kinase A (PKA) and exchange proteins directly activated by cAMP (EPAC1 and EPAC2, respectively) (*57*). Treatment of STC-1 cells with PKI-14-22 (100 nM), a selective PKA inhibitor, led to a clear trend (*P* = 0.104) toward a reduction in glucose-stimulated GIP release (Fig. 10B). Incubation of STC-1 cells with a selective inhibitor of EPAC1 [(R)-CE3F4; 10 μM] had no significant effect on glucose (20 mM)–stimulated GIP secretion (Fig. 10C). In contrast, incubation of STC-1 cells with a dual of EPAC1/2 (ESI-09; 10 μM) or a selective EPAC2 (ESI-05; 10 μM) inhibitor completely abolished glucose-induced GIP release (Fig. 10, D and E). Treatment with either of these two inhibitors also strongly decreased forskolin-stimulated GIP secretion (Fig. 10, D and E). In agreement with these observations, a selective EPAC2 activator (Sp-8-BnT-cAMPS; alternative name: S220; 10 μM) (*58*) significantly increased GIP release in a glucose-like fashion (Fig. 10F).

qRT-PCR studies demonstrated that *Epac2* mRNA was expressed at much higher levels than *Epac1* mRNA in STC-1 cells (fig. S7A). Moreover, mining of published RNA sequencing (RNA-seq) data showed the presence of *Epac2/EPAC2* mRNA but no detection of *Epac1*/*EPAC1* transcripts in mouse (*54*) and human (*34*) K cells (fig. S7, B and C). Thus, the observation that EPAC2, but not EPAC1, plays a role in glucose-stimulated GIP release is most likely due to the predominant expression of EPAC2 in K cells (STC-1 cells).

cAMP assays showed that glucose (20 mM) treatment of STC-1 cells resulted in a significant increase in cytosolic cAMP levels (Fig. 10G). This effect was completely blocked by the selective cannabinoid receptor agonist, WIN55 (10 μM) (Fig. 10G), suggesting that the ability of WIN55 to interfere with glucose-stimulated GIP secretion is due to WIN55-induced reductions in cAMP levels.

### Agonist activation of other G_i_ receptors expressed by STC-1 cells also suppresses glucose-induced GIP secretion

We next studied whether activation of other G_i_-coupled receptors endogenously expressed by STC-1 cells could mimic the inhibitory effect of WIN55 on glucose-stimulated GIP secretion. Previous studies have shown that mouse K cells also express G_i_-coupled galanin (*59*) and somatostatin (SST) (*55*) receptors. On the basis of these findings, we treated STC-1 cells with galanin (100 nM), SST (100 nM), or WIN55 (10 μM; positive control), followed by GIP release measurements. We found that galanin and SST completely blocked glucose-induced GIP secretion, in a fashion similar to WIN55 (fig. S8). This observation suggests that activation of G_i_-coupled receptors in general interferes with glucose-stimulated GIP release from STC-1 cells.

### Agonist activation of G_s_-coupled GPCRs expressed by STC-1 cells stimulates GIP secretion

We next examined whether stimulation of G_s_-coupled receptors endogenously expressed by STC-1 cells resulted in changes in GIP release. We treated STC-1 cells with INT-777 (30 μM) (fig. S9A), an agonist for the G_s_-coupled bile acid receptor (GPBAR; former name: TGR5) (*60*), or 2-palmitoyl glycerol (2-PG; 100 μM), an agonist for the G_s_-coupled GPR119 receptor (*61*) (fig. S9B). We found that both agonists resulted in significant increases in GIP secretion. These stimulatory effects were greatly reduced by cotreatment of cells with ESI-05 (10 μM), a selective EPAC2 inhibitor (fig. S9, A and B), indicating that EPAC2 also plays a critical role in mediating G_s_-induced increases in GIP release in STC-1 cells.

### Activators of both PKA and EPAC2 stimulate GIP release from STC-1 cells

To explore the possibility that direct activators of PKA and EPAC1 might also promote GIP release, we incubated STC-1 cells with 8-pCPT-2-O-Me-cAMP-AM (1 μM), a selective EPAC1 activator, 8-bromo-cAMP (100 μM), a selective PKA activator, and S220 (10 μM), a selective EPAC2 activator (positive control). We found that treatment with the PKA and EPAC2 activators resulted in significant increases in GIP secretion, while the EPAC1 activator did not affect basal GIP release (fig. S10). This observation suggests that both EPAC2 and PKA contribute to enhanced GIP release caused by the activation of receptors that increase intracellular cAMP levels.

### WIN55 also suppresses glucose-induced GIP secretion in mouse duodenal organoids

To measure GIP release in a more physiological setting, we also carried out studies with mouse duodenal organoids. Treatment of mouse duodenal organoids prepared from wild-type (WT) mice with a high concentration of glucose (20 mM) led to a ∼2-fold increase in GIP secretion (fig. S11A). This effect was nearly completely abolished by cotreatment with WIN55 (10 μM) (fig. S11A). In agreement with the data obtained with STC-1 cells, the glucose-stimulated increase in GIP release from mouse duodenal organoids was absent in the presence of a selective EPAC2 inhibitor (ESI-05; 10 μM) (fig. S11B). In contrast, a selective inhibitor of EPAC1 [(R)-CE3F4; 10 μM] did not interfere with glucose stimulation of GIP release (fig. S11C), again consistent with the results obtained with STC-1 cells.

### In vivo studies involving the use EPAC2 knockout mice and a selective EPAC2 inhibitor

We next wanted to determine whether efficient glucose-stimulated GIP release also required EPAC2 activation in vivo. To address this question, we treated EPAC2 knockout (KO) and WT control mice (8-week-old males) with an oral glucose bolus (2 g/kg) and determined plasma GIP levels 15 min later. Under these experimental conditions, EPAC2 KO mice showed significantly reduced plasma GIP levels before and after oral glucose treatment as compared to WT control mice (fig. S11D). We observed similar reductions in plasma GIP levels with WT mice (8-week-old C57/BL/6 male mice) that had been pretreated with ESI-05, a selective EPAC2 inhibitor (3 mg/kg, ip, given 30 min before the oral glucose bolus) (fig. S11E). Together, these in vivo results, together with the in vitro data presented in the previous paragraphs, strongly support the concept that efficient glucose-stimulated GIP release requires the activation of K cell EPAC2.

## DISCUSSION

GLP-1 and GIP mimetics have emerged as highly efficacious drugs for the treatment of T2D and obesity (*12*–*14*). As a result, this has led to renewed efforts to identify signaling pathways capable of modulating the release of endogenous GLP-1 and GIP from enteroendocrine cells for therapeutic purposes.

In the present study, we focused on signaling mechanisms that modulate the release of GIP from mouse K cells in vivo. During the past few years, research in the GIP field has experienced a renaissance (*4*, *7*, *62*). This renewed interest is mainly due to the fact that the GLP-1/GIPR coagonist tirzepatide shows remarkable antidiabetic and antiobesity effects that exceed those of GLP-1–only agonists (e.g., semaglutide) in the approved doses (*16*, *17*, *19*, *20*).

GIP-secreting K cells express a large number of GPCRs that modulate GIP release (*7*, *33*, *34*). GPCRs are linked to one or more functional classes of heterotrimeric G proteins. By analyzing published RNA-seq data, we found that mouse and human K cells express several members (α subunits) of the G_i_ family (gene names: *Gnai1*, *Gnai2*, *Gnai3*, *Gnao1*, and *Gnaz*). These G protein α subunits are expressed by both mouse and human K cells (fig. S1). G_i_-type G proteins, following their activation by agonist-occupied GPCRs, are not only known to inhibit adenylyl cyclase but can also modulate the activity of various ion channels and other cellular signaling pathways (*37*). The potential roles of G_i_-type G proteins in regulating K cell function in vivo remain unexplored.

By using DREADD technology, we generated a mouse model (K-GiD mice) that enabled us to selectively activate G_i_-type G proteins in K cells in vivo. Studies with K-GiD mice showed that selective activation of G_i_ signaling in K cells inhibited GIP secretion in vivo (Fig. 1E). Selective activation of K cell G_i_ signaling also lowered glucose-induced increases in plasma GIP and insulin levels, resulting in significantly impaired glucose tolerance in both lean and obese mice (Figs. 2, B to D; and 4, F, I, and K). These data indicate that activation of K cell G_i_ signaling leads to impaired glucose homeostasis in vivo.

When K-GiD mice consumed a mixed meal (Ensure Plus), DCZ-induced activation of K cell G_i_ signaling caused significantly smaller increases in plasma insulin and GIP levels and impaired glucose tolerance as compared to control mice (Fig. 3, A to C). These data indicate that the detrimental effects on GIP release and glucose tolerance caused by activation of K cell G_i_ signaling can also be observed under more physiological conditions (ingestion of a mixed meal).

The ingestion of lipids leads to robust increases in plasma GIP levels (*4*, *45*). As discussed elsewhere, these effects are most likely mediated by K cell GPCRs that can be activated by long-chain fatty acids or monoacylglycerols (e.g., GPR119, GPR120, and FFAR1) (*4*, *45*). While oral administration of olive oil resulted in a pronounced increase in plasma GIP levels in control mice, this effect was almost completely abolished after DCZ-induced activation of K cell G_i_ signaling in K-GiD mice (Fig. 3H). In sum, these data indicate that stimulation of K cell G_i_ signaling in vivo greatly reduced GIP secretion under all experimental conditions used in the present study (administration of oral glucose, a mixed meal, or oral olive oil).

Since activation of K cell G_i_ signaling interferes with GIP secretion and causes impaired glucose homeostasis in vivo, we tested the hypothesis that inhibition of K cell G_i_ signaling in vivo might lead to elevated plasma GIP levels and improved glucose tolerance. To test this hypothesis, we generated a mouse strain that expressed the S1 catalytic subunit of PTX selectively in K cells (K-Gi-KO mice). PTX inactivates the α subunits of G_i_-type G proteins (except for Gα_z_) via adenosine diphosphate (ADP) ribosylation of a C-terminal cysteine residue (*37*). Consistent with our hypothesis, K-Gi-KO mice displayed elevated plasma GIP levels under basal conditions (Fig. 5G). Similarly, K-Gi-KO mice exhibited increased plasma GIP levels and improved glucose homeostasis after oral treatment with glucose, olive oil, or a mixed meal (Ensure Plus) (Figs. 6 and 7). These data convincingly demonstrated that the inhibition of K cell G_i_ signaling greatly enhances GIP secretion in vivo, which, in turn, has a positive impact on glucose homeostasis.

To explore the potential translational relevance of these findings, we maintained K-Gi-KO mice and their control littermates on an HFD to induce obesity and deficits in glucose homeostasis including impaired glucose tolerance. Under these conditions, K-Gi-KO mice showed significantly improved oral glucose tolerance as compared to obese control mice (Fig. 8E). This beneficial metabolic effect could be blocked by treatment with a GIPR antagonist (GIPA-2) (Fig. 8F) (*49*), strongly suggesting that the observed improvement in glucose tolerance involves the stimulation of β cell GIPRs. Obese K-Gi-KO mice also displayed considerably smaller blood glucose excursions than their obese control littermates (Fig. 9A). Together, these findings suggest that inhibition of K cell G_i_ signaling may prove clinically useful for treating deficits in glucose homeostasis.

In a related study, we recently generated and analyzed a mouse strain that selectively expressed a G_s_-coupled designer receptor (G_s_ DREADD) in K cells (*31*). We demonstrated that stimulation of K cell G_s_ signaling led to enhanced GIP secretion, which, in turn, resulted in improved glucose homeostasis in obese mice and in a mouse model of T2D (*31*). In another study, Lewis *et al.* (*32*) used a chemogenetic strategy to express a G_q_-coupled designer receptor (G_q_ DREADD) in K cells. The authors found that stimulation of K cell G_q_ signaling, similar to activation of K cell G_s_ signaling, resulted in a robust increase in plasma GIP levels and improved glucose tolerance (*32*). While chronic K cell G_q_ signaling also led to a decrease in food intake and body weight, these effects were not observed with K-Gi-KO mice (present study) or after chronic activation of K cell G_s_ signaling (*31*). The cellular and molecular mechanisms underlying these phenotypic differences remain to be elucidated in future studies.

Published RNA-seq data indicate that both mouse and human K cells express a considerable number of G_i_-coupled GPCRs at moderate to high levels (fig. S12). As expected, both mouse and human K cells express various SST receptor subtypes. Somewhat unexpectedly, most other G_i_-coupled receptors expressed by mouse K cells were not expressed by human K cells and vice versa (fig. S12). For example, human K cells, but not mouse K cells, express several biogenic amine receptors, including different α_2_-adrenergic receptor subtypes as well as D_4_ dopamine and M_4_ muscarinic acetylcholine receptors (fig. S12). Since biogenic amine receptors are the target of numerous drugs in current clinical use (*35*), it is possible that pharmacological blockade of one or more of these receptors could promote GIP release and improve impaired glucose homeostasis. We are planning to test this hypothesis in a follow-up study using human duodenal organoids.

As shown in fig. S12A, mRNA encoding the CB_1_ receptor was particularly abundant in mouse K cells. By using the CB_1_ receptor as a model system, we carried out additional in vitro studies using GIP-producing STC-1 cells and mouse duodenal organoids to further explore the molecular pathways linking the activation of a prototypic G_i_-coupled receptor to inhibition of GIP release. Glucose-induced GIP release from STC-1 cells was blocked by WIN55, a selective cannabinoid receptor agonist (Fig. 10A), and glucose treatment resulted in a significant increase in intracellular cAMP levels, a response that could be completely blocked by WIN55 (Fig. 10G). These findings are in agreement with the observation that CB_1_ receptor agonism reduces GIP release from primary duodenal mouse cultures and in response to an oral glucose gavage in vivo (*55*). On the other hand, treatment of rats with a CB_1_ receptor antagonist (AM251) raised basal plasma GIP levels, suggesting that GIP secretion is suppressed by a tonic cannabinoid tone (*55*). Endogenous cannabinoid receptor agonists are found throughout the gastrointestinal tract (*63*).

To explore whether other G_i_-coupled receptors endogenously expressed by STC-1 cells could mimic the inhibitory effect of WIN55 on glucose-stimulated GIP secretion, we treated STC-1 cells with galanin and SST. These two agonists, which activate G_i_-coupled galanin and SST receptors, respectively, completely blocked glucose-induced GIP release, in a fashion similar to WIN55 (fig. S8), suggesting that activation of G_i_-coupled receptors in general interferes with glucose-stimulated GIP secretion.

cAMP exerts its cellular actions via binding to two major cAMP binding proteins, PKA and EPAC1/2 (*64*). Studies with STC-1 cells showed that glucose-induced GIP release was blocked by a selective EPAC2 inhibitor (ESI-05) (Fig. 10E) and that a selective EPAC2 activator (S220) stimulated GIP secretion (Fig. 10F). In agreement with these findings, glucose failed to enhance GIP release from mouse duodenal organoids in the presence of ESI-05 (fig. S11B). These data indicate that efficient glucose stimulation of GIP release requires cAMP-dependent EPAC2 signaling.

However, in vivo studies showed that glucose-induced increases in plasma GIP levels were only partially reduced in mice lacking EPAC2 (EPAC2 KO mice) or after treatment of WT mice with a selective EPAC2 inhibitor (fig. S11, D and E). These data suggest that other signaling molecules or pathways, besides EPAC2, contribute to glucose-stimulated GIP secretion in vivo. In agreement with this concept, studies with STC-1 cells demonstrated that a selective PKA activator was able to stimulate GIP secretion (fig. S10) and that a low concentration of a selective PKA inhibitor showed a clear trend toward reducing glucose-dependent GIP release (Fig. 10B). These data support a model in which EPAC2 and PKA act in synergy to mediate cAMP-dependent increases in GIP release from K cells.

Similar to EPAC2, EPAC1 is also activated by elevated intracellular cAMP levels. However, *Epac1* is expressed at considerably lower levels than *Epac2* in STC-1 cells, and *Epac1*/*EPAC1*, in contrast to *Epac2*/*EPAC2*, mRNA could not be detected in mouse (*54*) and human (*34*) K cells in RNA-seq studies (fig. S7). Thus, our finding that EPAC2, but not EPAC1, plays a role in glucose-stimulated GIP release is most likely due to the predominant expression of EPAC2 in K cells (STC-1 cells).

It should be noted in this context that EPAC2 is known to play a central role in the release of other metabolically important hormones including insulin (*65*, *66*). In pancreatic β cells, for example, EPAC2 enhances GSIS by stimulating a signaling cascade involving Rap1 guanosine triphosphatase and phospholipase C–ε, ultimately leading to calcium mobilization and increased insulin release (*65*, *66*). More detailed studies are required to explore whether a similar mechanism is operative in enteroendocrine K cells.

We also treated STC-1 cells with agonists targeting two G_s_-coupled receptors (GPR119 and GPBAR) endogenously expressed by K cells (*31*). GPR119 is activated with high potency by monoacylglycerols derived from the breakdown of triglycerides (*61*, *67*). Treatment of STC-1 cells with both agonists caused significant increases in GIP secretion, and these effects were strongly reduced by cotreatment with a selective EPAC2 inhibitor (fig. S9, A and B). This observation suggests that EPAC2 plays a general role in promoting GIP release following the activation of GPCRs capable of elevating intracellular cAMP levels.

In the present study, we measured plasma GLP-1 levels in the absence of any agents that prevent GLP-1 degradation. However, since reliable measurements of plasma GLP-1 levels in mice require treatment with inhibitors of dipeptidyl peptidase 4 and neprilysin (*68*), the GLP-1 data reported in this study are of limited value. Since these enzymatic inhibitors also affect the plasma levels of other metabolically important peptides, we did not treat mice with enzymatic inhibitors in this study.

A key question arising from the present study is to which extent our findings are of potential translational relevance. One potential strategy aimed at enhancing GIP secretion could be the development of gut-targeted antagonists for G_i_-coupled receptors expressed by K cells. To achieve high concentrations of these agents near the gastrointestinal epithelium and not in other tissues, these agents must be designed to be poorly absorbed from the gastrointestinal tract. This type of approach has recently been applied successfully to enhance incretin secretion after oral administration of a combination of gut-restricted GPR119 and GPR40 (FFAR1) agonists in both mice and humans (*69*).

In conclusion, the use of K cell–specific mouse models allowed us to identify the in vivo metabolic roles of G_i_-type G proteins endogenously expressed by K cells. A better understanding of the G protein signaling pathways regulating GIP release may stimulate the development of therapeutic approaches useful for the treatment of conditions characterized by impaired glucose homeostasis.

### Limitations of the study

To further confirm the translational relevance of our findings, we are planning to explore whether the signaling pathways identified in the present study are also operative in human K cells. Moreover, in future in vivo studies, we will test antagonists that are able to block G_i_-coupled receptors endogenously expressed by K cells. Since essentially all G_i_-coupled receptors are expressed in multiple tissues, we will focus on targeting receptors that are enriched in K cells as compared to other cell types. The use of DREADDs is associated with certain caveats. DREADDs are often expressed at levels that exceed those of endogenous GPCRs, which can lead to altered signaling or physiological outcomes. In addition, it is unclear in most cases how closely DREADDs mimic the cellular localization and interaction with regulatory proteins displayed by native GPCRs.

## MATERIALS AND METHODS

### Study approval

All animal studies were carried out according to the National Institutes of Health (NIH) Guide for the Care and Use of Laboratory Animals and approved by the National Institute of Diabetes and Digestive and Kidney Diseases (NIDDK) Animal Care and Use Committee (NIH, Bethesda, MD) (protocol no. ASP K005-LBC-24).

### Mouse maintenance

Mice were group housed at 23°C and fed ad libitum on a 12-hour light/12-hour dark cycle. Most studies were carried out with mice consuming a regular chow diet (#5018, LabDiet; energy density, 3.05 kcal/g). In a subset of experiments, mice were maintained on an HFD (F3282, Bioserv; 60% kcal from fat, energy density, 5.5 kcal/g) after reaching 8 weeks of age. Metabolic studies were performed with male mice that were at least 8 weeks old.

### Generation of K cell–specific G_i_ mutant mice

To generate mice selectively expressing a GiD (*38*) in K cells (K-GiD mice), we crossed *Rosa26-LSL-hM4Di* mice (alternative name: LSL-GiD mice) (*40*) with *Gip-Cre* mice that expressed Cre recombinase under the transcriptional control of the *Gip* promoter (*41*). Cre-positive hemizygous *LSL-hM4Di* mice (K-GiD mice) are predicted to express the GiD designer receptor selectively in K cells. *Rosa26-LSL-hM4Di* mice lacking the *GIP-Cre* transgene served as control animals throughout the study (genetic background of control and K-GiD mice: C57BL/6).

To generate mice lacking functional G_i_ proteins selectively in K cells (K-Gi-KO mice), we used *ROSA26^PTX/PTX^* mice generated by Regard *et al.* (*42*). These mice express the S1 catalytic subunit of PTX (S1 PTX), which inactivates Gα subunits of the G_i/o_ family (except for Gα_z_) in a Cre-dependent fashion (*42*,*47*,*48*). We crossed *ROSA26^PTX/PTX^* mice with *GIP-Cre* mice. Cre-positive hemizygous *ROSA26^PTX^* mice expressed S1 PTX selectively in K cells (K-Gi-KO mice), while littermates not expressing *Cre* served as control animals. Mouse tail DNA was used for PCR genotyping of the different mutant mouse strains. PCR reactions were carried out using standard protocols. Primer sequences are listed in table S1. All mice used for these matings had been backcrossed at least seven times onto the C57BL/6 background.

### Physiological studies

Body composition (lean and fat mass) was determined via EchoMRI (EchoMRI100H, EchoMRI LLC). In acute DCZ challenge tests, K-GiD mice and control littermates received a single dose of DCZ [10 μg/kg in phosphate-buffered saline (PBS) via oral gavage or intraperitoneal injection, as indicated]. Before DCZ treatment, mice had free access to food or had been fasted for 6 hours, as indicated in the legends for Figs. 1 to 4. For chronic DCZ administration studies, DCZ was added to the drinking water at a concentration of 10 mg/liter. Oral glucose and mixed meal tolerance tests were conducted after a 6-hour fast. For these tests, mice received either an oral bolus of glucose (2 g/kg for chow-fed mice and 1 g/kg for HFD mice) or Ensure Plus (10 ml/kg; Abbott, IL), respectively, either without or with oral DCZ (10 μg/kg). Blood glucose levels were measured immediately before glucose or Ensure Plus administration and at defined posttreatment time points. To study GSIS, we treated mice with glucose via oral gavage (2 g/kg for chow-fed mice and 1 g/kg for HFD mice) either without or with oral DCZ (10 μg/kg). Blood was collected immediately before and at defined posttreatment time points for the measurement of plasma incretin, insulin, and glucagon levels. For insulin tolerance tests, mice that had been fasted for 4 hours were injected intraperitoneally with Humulin R (0.75 U/kg for chow-fed mice and 1.5 U/kg for HFD mice) either without or with DCZ (10 μg/kg), administered either orally or intraperitoneally, as indicated. Blood glucose levels were measured immediately before and at specific postinjection time points. Acute food intake was measured using single-housed mice by manually weighing food pellets. After a 24-hour fast, mice had free access to food (regular chow or HFD) for a 2-hour period. All mice received oral DCZ (10 μg/kg) 30 min before food intake studies. For refeeding studies, food was withdrawn at the beginning of the onset of the dark cycle for 24 hours, followed by refeeding for 2 hours.

### Olive oil challenge test

Mice that had been fasted for 6 hours received an oral gavage of olive oil (10 μl/g of body weight; Sigma-Aldrich). Blood was collected before and 60 min after oil administration for blood glucose and plasma hormone measurements.

### STZ-induced diabetes

To generate a mouse model of severe T2D, 8-week-old male K-Gi-KO mice and control littermates received daily injections of STZ (#S0130, MilliporeSigma) or vehicle for 5 consecutive days (50 mg/kg, ip), as described previously (*50*).

### In vivo studies with EPAC2 KO mice

EPAC2 KO mice (*70*) (genetic background: C57/BL/6) were provided by X. Chen and O. Pochynyuk (The University of Texas Health Science Center, Houston, TX). Age-matched WT mice with the same genetic background were used as control animals. Following a 6-hour fast, mice received an oral bolus of glucose (2 g/kg). Blood was collected from the tail vein immediately before and at defined posttreatment time points for the measurement of blood glucose and plasma GIP levels.

### Determination of blood glucose and plasma hormone levels

Blood glucose was measured using an automated blood glucose meter (Glucometer Elite Sensor, Bayer). Blood samples were collected from the tail vein and transferred into EDTA-coated tubes containing a protease inhibitor cocktail (cOmplete tablets, Roche) and a dipeptidyl peptidase 4 inhibitor (KR-62436; 100 μM). Plasma was obtained by centrifugation of blood at 10,000*g* for 10 min at 4°C. Plasma levels of insulin (#90080, Crystal Chem), total GIP (#81527, Crystal Chem), and glucagon (#81518, Crystal Chem) were determined using hormone-specific enzyme-linked immunosorbent assay (ELISA) kits, according to the manufacturer’s protocols.

### Cell culture

STC-1 cells (CRL-3254, American Type Culture Collection) were cultured in high-glucose Dulbecco’s modified Eagle’s medium (DMEM; Gibco). DMEM was supplemented with 10% fetal bovine serum (Gibco) and a 1× penicillin-streptomycin solution (Life Technologies). Cells were passaged every 3 days at ∼80 to 90% confluence. All cells were cultured in a humidified incubator at 37°C in a 5% CO_2_ atmosphere in T75 flasks.

### Mouse duodenal organoids

Isolated duodenal crypts were cultured as previously described, with minimal modifications (*71*). Briefly, crypts were released from the murine duodenum and incubated at 4°C for 30 min in Dulbecco’s PBS (without calcium and magnesium) containing 2 mM EDTA (Crystalgen, NY). After incubation in PBS plus EDTA, mouse duodenal tissues were transferred to ice-cold PBS. Tissues were then shaken vigorously for 1 min to obtain a crypt-enriched fraction. After size fractionation using 100- and 70-μm filters, crypts were collected and embedded in extracellular matrix gel from Engelbreth-Holm-Swarm murine sarcoma (Sigma-Aldrich, MO). The crypts were then placed into 24-well plates (300 crypts per 60 μl of Matrigel per well). IntestiCult mouse organoid growth medium (STEMCELL Technologies) was used as the culture medium. Media were changed every 2 or 3 days, and organoids were passaged 5 to 7 days after seeding.

### In vitro GIP secretion studies

STC-1 cells (seeding density, 0.05 × 10^6^ cells) (cell passages 15 to 30) or duodenal crypts (300 to 400 crypts per 40 μl of Matrigel per well) were plated into 24-well plates (#3524, Corning) with high-glucose DMEM 18 to 24 hours before hormone release studies. On the next day, cells (∼80% confluent) or organoids (passaged twice prior to treatments) were washed with Krebs-Ringer solution (Hepes-buffered; Thermo Fisher Scientific), followed by a 20-min preincubation with selective agonists or inhibitors [WIN55 (MedChemExpress), PKI-14-22 (MedChemExpress), ESI-05 (MedChemExpress), (R)-CE3F4 (MedChemExpress), S220 (BIOLOG Life Sciences), gallein (MedChemExpress), 8-pCPT-2-O-Me-cAMP-AM (MedChemExpress), 8-bromo-cAMP (MedChemExpress), galanin (Alomone Labs), SST (Sigma-Aldrich), INT-777 (Cayman Chemical), and 2-PG (Cayman Chemical)]. Subsequently, cells or organoids were incubated for 3 hours with 20 mM glucose, 10 μM forskolin (Cayman Chemical), ESI-09 (MedChemExpress), WIN55, PKI-14-22, ESI-05, (R)-CE3F4, or S220. Supernatants were obtained and centrifuged (1500*g* at 4°C for 5 min) to remove any floating cells or debris. The resulting supernatants were transferred to fresh Eppendorf tubes, and samples were either directly processed for GIP measurements while kept on ice or stored at −20°C until further use. Last, cells or organoids were lysed with radioimmunoprecipitation assay buffer, and total protein content was determined using the Pierce BCA protein assay kit (#PI23228, Thermo Fisher Scientific). Total GIP was determined via ELISA (#81527, Crystal Chem), and data were normalized by protein content. All experiments were done in triplicate.

### cAMP assay

cAMP concentrations in cultured STC-1 cells were determined using the direct cAMP ELISA kit (#ADI-900-066, Enzo). Culturing conditions are described in the previous paragraph. Initially, STC-1 cells were treated with 20 mM glucose with or without 10 μM WIN55 for 3 hours at 37°C. At the end of this incubation period, supernatant from each treated well was aspirated, and cells were scraped into lysis buffer (0.1 M HCl) and lysed via syringe disruption (25 gauges). Lysed samples were then centrifuged (1500*g* at 4°C for 5 min) to remove any floating cells or debris. The resulting supernatants were transferred to fresh Eppendorf tubes and directly processed for cAMP measurements while kept on ice. cAMP data were normalized by total protein content determined by a Pierce BCA protein assay (#PI23228, Thermo Fisher Scientific). All experiments were carried out in quadruplicate. In each individual experiment, cAMP levels were expressed relative to cAMP levels measured with vehicle-treated control cells.

### Immunohistochemistry

Mouse intestinal tissues were fixed overnight in 4% paraformaldehyde, processed using ethanol and xylene, and then embedded into paraffin blocks. For immunofluorescence staining studies, deparaffinized slides were heated in IHC-Tek epitope retrieval buffer (Invitrogen) for 30 min at 98°C and blocked and permeabilized in PBS containing 5% goat serum and 0.25% of Triton X-100. Slides with intestinal tissues were incubated overnight at 4°C with a mixture of mouse anti-HA primary antibody (1:1000; #2367, Cell Signaling Technology) or anti-myc primary antibody (1:1000; #2276S, Cell Signaling Technology) and rabbit anti-GIP antibody (1:1000; #ab209792, Abcam). After several washing steps, slides were incubated for 1 hour at room temperature with a mixture of Alexa Fluor 594– and Alexa Fluor 488–conjugated secondary antibodies (1:500; #A-11012 and A-11001, respectively, Thermo Fisher Scientific). Images were obtained using a confocal microscope (LSM700, Zeiss). To determine the percentage of GIP-expressing K cells that were HA- or myc-positive, we analyzed duodenal slices from five different K-GiD and K-Gi-KO mice.

### qRT-PCR analysis of gene expression

Total RNA was extracted from frozen tissues or cultured cells using the Direct-Zol micro-prep kit (Zymo Research), and SuperScript III First-Strand Synthesis SuperMix (Invitrogen) was used to prepare cDNA. The SYBR green method (Vazyme) was used to perform quantitative PCR studies. Gene expression data were normalized relative to the expression of *m36b4* using the ΔΔ*C*_t_ method. All PCR primers used in this study are listed in table S1.

### Intestinal incretin content

To measure total intestinal content of GIP, the entire mouse intestinal tract was excised from the beginning of the duodenum to the end of the ileum following CO_2_ euthanasia, washed with PBS, weighed, and homogenized mechanically with an Ika Ultra-Turrax device (Sigma-Aldrich) in 74% ethanol containing 0.15 M HCl. After an overnight incubation at 4°C, the homogenate was centrifuged at 12,000*g* for 5 min at 4°C. The supernatant was diluted 1000-fold with ELISA kit diluent buffer for the measurement of GIP concentrations via ELISA.

### Pancreatic hormone content

To determine mouse pancreatic insulin and glucagon content, whole pancreata were first weighed and then homogenized in acid-ethanol (1.5% HCl in 70% ethanol and 3 ml per pancreas) using a Precellys Evolution Touch homogenizer (Bertin Instruments), followed by a 1-hour incubation on ice. Tissue homogenates were centrifuged at 10,000*g* for 20 min at 4°C, and insulin and glucagon levels were measured in the supernatant using hormone-specific ELISA kits (see the “Determination of blood glucose and plasma hormone levels” section).

### Statistics

Data are expressed as the means ± SEM for the indicated number of observations. Data were assessed for statistical significance by one-way or two-way analysis of variance (ANOVA), followed by the indicated post hoc tests, or by two-tailed, unpaired Student’s t test, as appropriate. A *P* value of less than 0.05 was considered statistically significant. The specific statistical tests that were used are indicated in the figure legends.

## Supplementary Materials

### This PDF file includes:

Figs. S1 to S12 Table S1 References
