# Supplementary material for: Altered Gi signaling in enteroendocrine K cells in vivo causes pronounced changes in glucose homeostasis
Source: Sci Adv. 2026 Jul 29;12(31):eaeb9805. doi: 10.1126/sciadv.aeb9805 (PMC13418537; doi:10.1126/sciadv.aeb9805)
Supplement: Supplementary file 1 — Figs. S1 to S12 Table S1 References [file sciadv.aeb9805_sm.pdf]

Science Advances
AAAS

Supplementary Materials for

Altered G_i signaling in enteroendocrine K cells in vivo causes pronounced changes in glucose homeostasis

Osvaldo Rivera-Gonzalez *et al.*

Corresponding author: Jürgen Wess, jurgenw@niddk.nih.gov

Sci. Adv. **12**, eaeb9805 (2026)
DOI: 10.1126/sciadv.aeb9805

This PDF file includes:

Figs. S1 to S12
Table S1
References

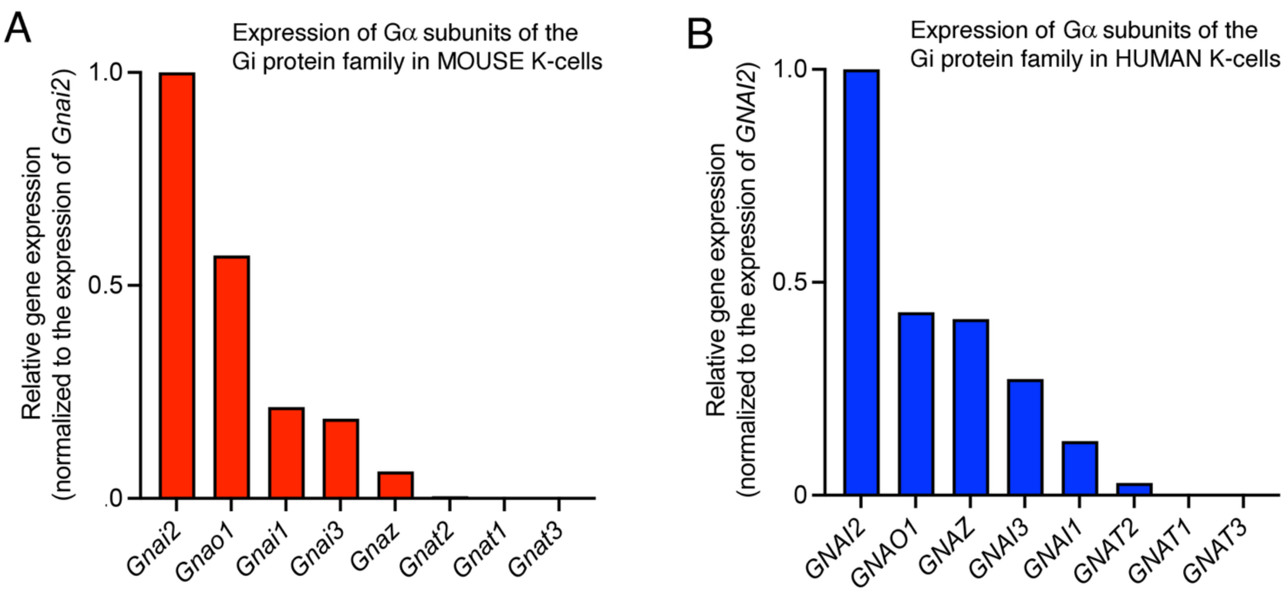

Fig. S1. Expression of Gα subunits of the G_i protein family in mouse and human K-cells. (**A**) Mouse K-cell transcript levels. (**B**) Human K-cell mRNA levels. The data shown were compiled from published data. Mouse gene expression data: (54). Human gene expression data: (34).

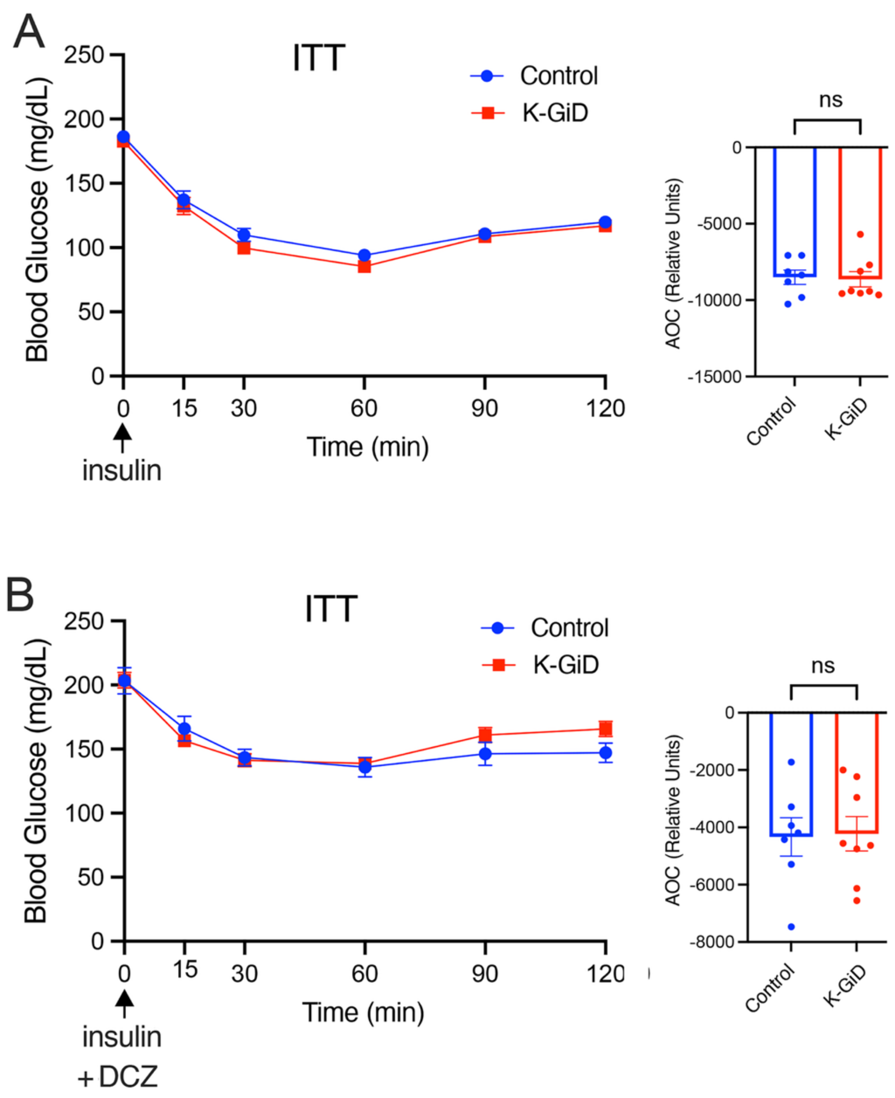

Fig. S2. Activation of K-cell G_i signaling does not affect insulin tolerance in K-GiD mice. (A, B) Insulin tolerance tests (ITT). After a 4-hr fast, K-GiD mice and control littermates were treated with an insulin injection (0.75 U/kg i.p.) alone (**A**) or co-treated with insulin (0.75 U/kg i.p.) plus DCZ (10 μg/kg i.p.) (**B**). Blood glucose levels were then determined at the indicated time points. AOC values are given as quantitative measures of the experimental data shown in panels (**A**) and **(B**). All experiments were carried out with male mice (age:10-11 weeks). Data are given as means ± SEM (n = 7 or 8 mice/group). AOC data were analyzed via two-tailed Student's t-test. AOC, area of the curve.

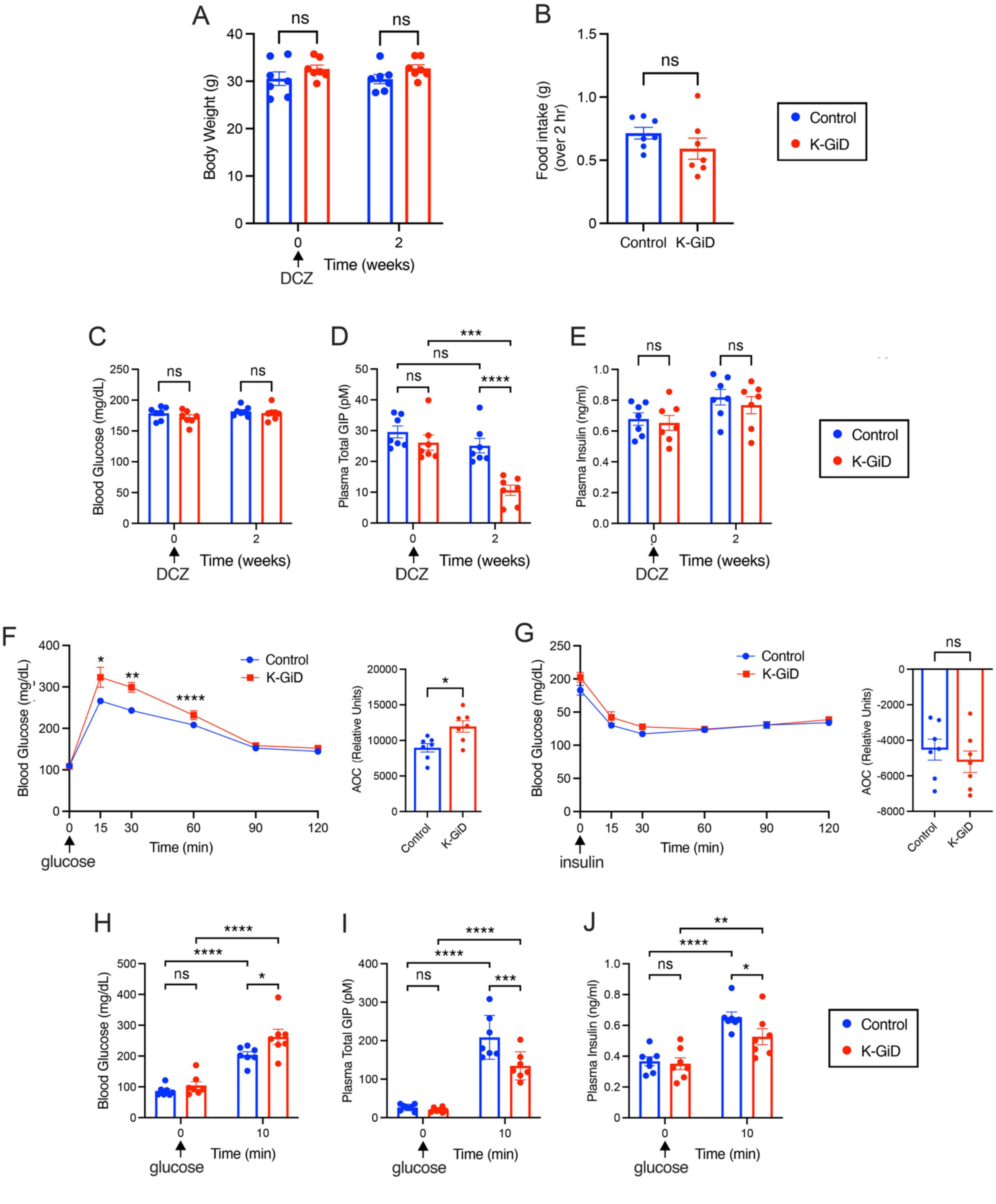

Fig. S3. Chronic activation of K-cell G_i signaling impairs glucose tolerance in lean K-GiD mice. (A) Body weight of K-GiD and control littermates maintained on DCZ drinking water for

2 weeks (mouse age: 20 weeks). **(B)** Food intake studies (regular chow). After a 24-hr fast, K-GiD and control mice were given free access to food for 2 hr. Mice had been consuming DCZ drinking water for two weeks. **(C-E)** Blood glucose and plasma hormone levels in mice after consumption of DCZ water for two weeks. Blood glucose (**C**), plasma GIP (**D**), and plasma insulin (**E**) were measured at the indicated time points. (**F**) OGTT. K-GiD mice and control littermates that had been consuming DCZ water for two weeks received an oral glucose bolus (2 g/kg). (**G**) ITT. K-GiD and control mice maintained on DCZ water for two weeks were injected with insulin **(**0.75 U/kg i.p.). **(H-J)** Blood glucose levels and plasma hormone levels in K-GiD and control mice treated with oral glucose (2 g/kg) after DCZ water consumption for two to three weeks. Blood glucose (**H**), plasma GIP (**I**), and plasma insulin (**J**) levels were measured at the indicated time points. All experiments were carried out with male mice (~20 weeks old) after a 6-hr fast except for ITT studies (4-hr fast). Data are given as means ± SEM (n = 7 or 9 mice/group). *P<0.05, **P<0.01, ***P<0.01, ****P<0.0001, as compared with the corresponding control group (2-way ANOVA followed by Tukey post-hoc analysis (**A, C-J**) or two-tailed Student's t-test (**B, F, G;** AOC data)). AOC, area of the curve; ns, no statistically significant difference.

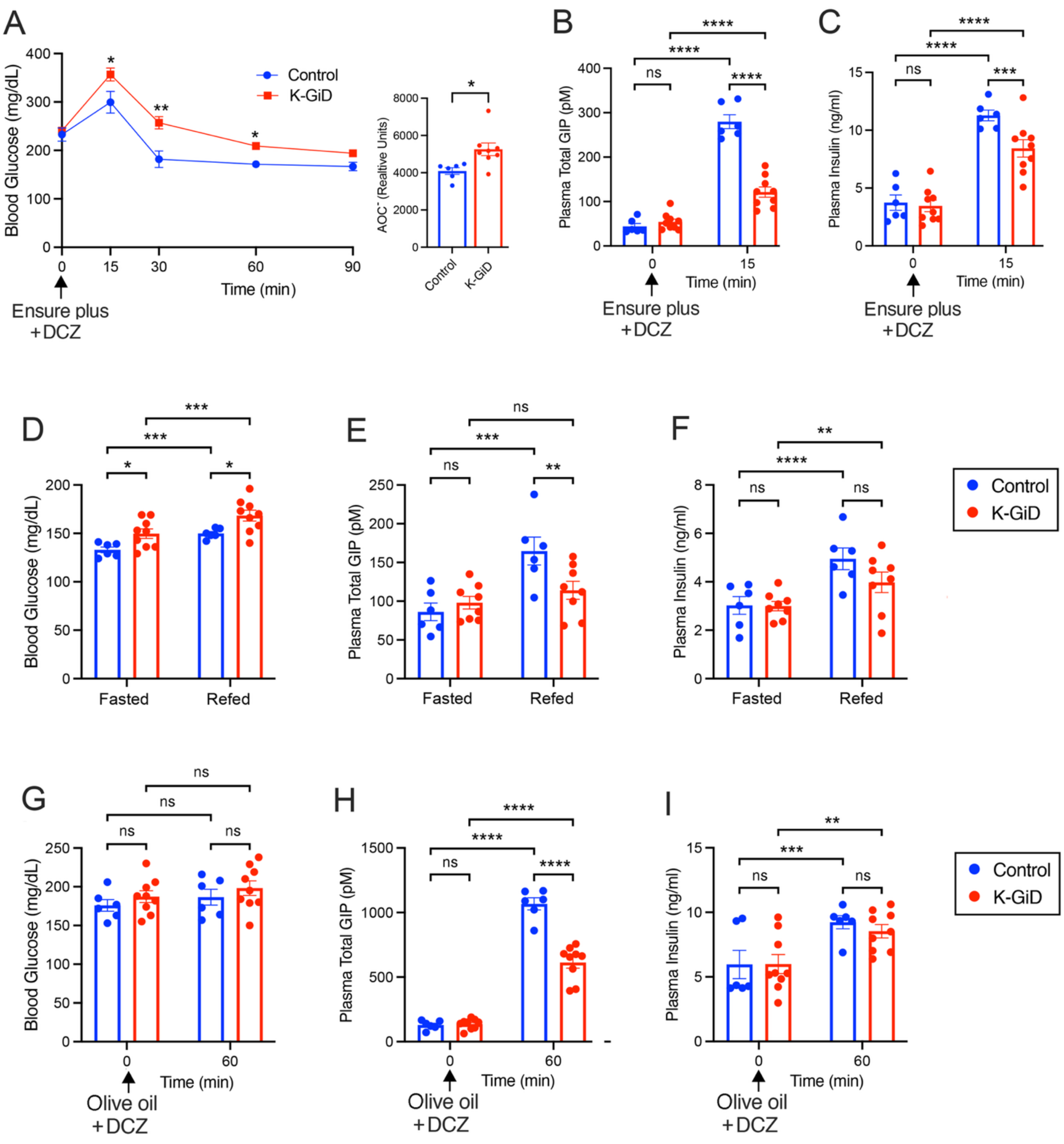

Fig. S4. Stimulation of K-cell G_i signaling in obese mice causes metabolic deficits in response to a mixed meal, refeeding, or olive oil ingestion. (A) Mixed meal (Ensure Plus) tolerance test. K-GiD mice and control littermates that had been maintained on a HFD for 15 weeks received an oral bolus of Ensure Plus (10 ml/kg) supplemented with DCZ (10 µg/kg), followed by the monitoring of blood glucose levels. **(B, C)** Measurement of plasma hormone

levels**.** HFD K-GiD and control mice were treated with oral Ensure Plus (10 ml/kg) supplemented with DCZ (10 μg/kg). Plasma GIP (**B**) and insulin (**C**) levels were measured at the indicated time points. **(D-F)** Refeeding studies. After a 24-hr fast, HFD K-GiD and control mice had free access to regular chow (higher carbohydrate content) for 2 hr. Blood glucose (**D**), plasma GIP (**E**), and plasma insulin (**F**) levels were measured immediately prior to and after refeeding. **(G-I)** Olive oil-induced changes in blood glucose and plasma hormone levels. After a 6-hr fast, HFD K-GiD and control mice received an oral bolus of olive oil (10 ml/g) supplemented with DCZ (10 μg/kg). Blood glucose (**G**), plasma GIP (**H**), and plasma insulin (**I**) levels were measured 60 min later. All experiments were carried out with male mice (age: 23-25 weeks) maintained on the HFD for at least 8 weeks. Data are given as means ± SEM (control mice, n = 6; K-GiD mice, n = 9). *P<0.05, **P<0.01, ***P<0.01, ****P<0.0001, as compared with the corresponding control group (2-way ANOVA followed by Tukey post-hoc analysis (**A-I**) or two-tailed Student's t-test (**A**; AOC bars), respectively). AOC, area of the curve; ns, no statistically significant difference.

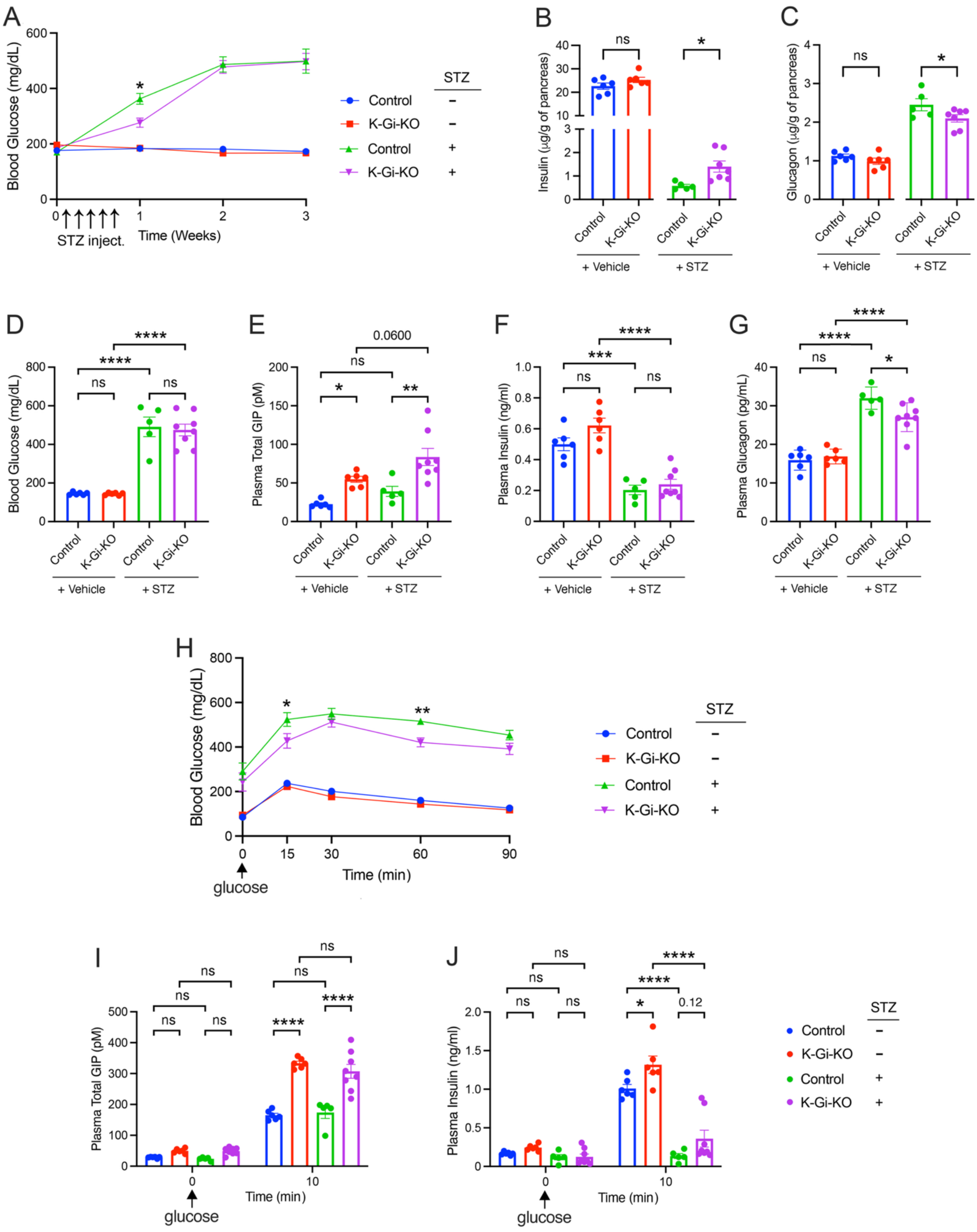

Fig. S5. **Effect of deficient K-cell G_i signaling on STZ-induced hyperglycemia. (A)** Blood glucose levels prior to and after STZ treatment. K-Gi-KO mice and control littermates were

injected with five consecutive doses of STZ (50 mg/kg i.p.), as described in detail under Materials and Methods. (**B, C**) Pancreatic insulin **(B)** and glucagon **(C)** content before and after treatment with STZ (4 weeks after the last STZ injection). **(D-G)** Blood glucose and plasma hormone levels after vehicle or STZ treatment. Blood glucose **(D)** and plasma GIP **(E)**, insulin **(F)**, and glucagon **(G)** levels were measured 4 weeks after STZ or vehicle treatment. **(H)** OGTT. All groups of mice received an oral glucose bolus (2 g/kg), followed by the monitoring of blood glucose levels. (**I, J**) Plasma hormone levels after administration of an oral glucose bolus (2 g/kg). Plasma GIP (**I**) and insulin (**J**) levels were measured at the indicated time points. All experiments were carried out with male mice after a 12-hr fast. Data are given as means ± SEM (n = 5-8 mice/group). *P<0.05, **P<0.01, ***P<0.01, ****P<0.0001, as compared with the corresponding control group (2-way ANOVA followed by Tukey post-hoc analysis (**A, D-J)** or two-tailed Student's t-test (**B, C**), respectively). ns, no statistically significant difference.

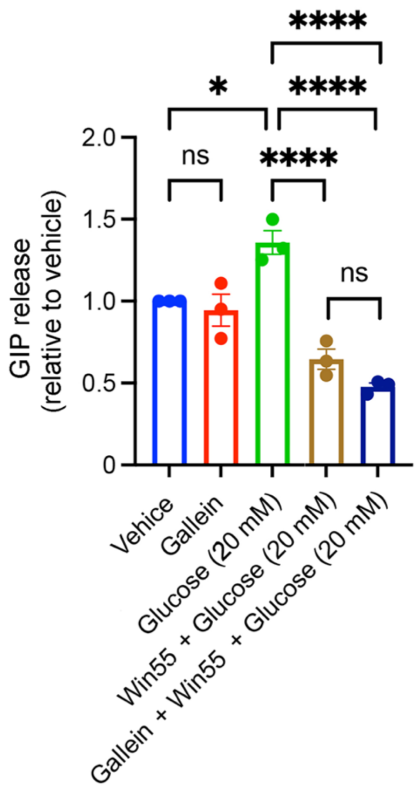

Fig. S6. Incubation of STC-1 cells with gallein does not affect WIN55-mediated suppression of glucose-induced GIP release. Treatment of STC-1 cells with gallein (10 μM), a selective inhibitor of βγ-mediated signaling, does not interfere with WIN55-dependent impairment of glucose-induced GIP release. Data are given as means ± SEM from three independent experiments. *P<0.05, ****P<0.0001 (1-way ANOVA followed by Tukey post-hoc analysis), as compared with the corresponding control group. ns, no statistically significant difference.

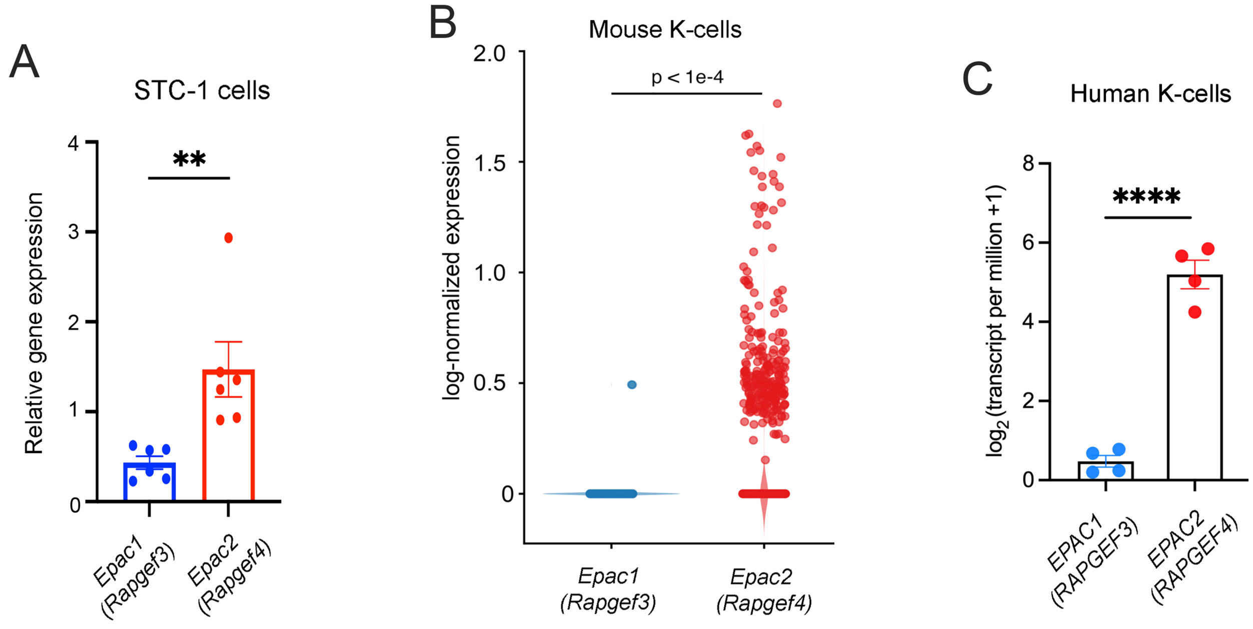

Fig. S7. Transcript levels of *Epac1/EPAC1* and *Epac2/EPAC2* in STC-1 cells and mouse and human K-cells. (A) *Epac2 (Rapgef4)* expression is significantly higher than *Epac1 (Rapgef3)* expression in STC-1 cells. Normalized gene expression data obtained via qRT-PCR are shown. (**B**) scRNAseq analysis of *Epac1 (Rapgef3)* and *Epac2 (Rapgef4)* transcript levels in K-cells from the mouse upper small intestine. The violin plot shows the distribution of log-normalized expression values for the two genes in mouse K-cells (n = 1055) (54). Each dot represents the transcript levels in one cell. Expression values were log-normalized using Seurat's standard log-transformation to account for differences in sequencing depth. The violins illustrate overall expression distributions, while jittered points highlight cell-to-cell variability. Significance was assessed using the Wilcoxon rank-sum test. (**C**) Expression levels of *EPAC1 (RAPGEF3)* and *EPAC2 (RAPGEF4)* in K-cells from human duodenal organoids. The plot shows $\log_2$ (TPM + 1) expression values for the two genes across GIP-Venus positive cells from bulk RNAseq studies (n = 4 biological replicates) (34). Individual dots represent expression values from each sample replicate. Gene expression values were log-transformed to stabilize variance. In (**A**) and (**C**), data are given as means ± SEM. **P<0.01, ****P<0.0001 (two-tailed Student's t-test).

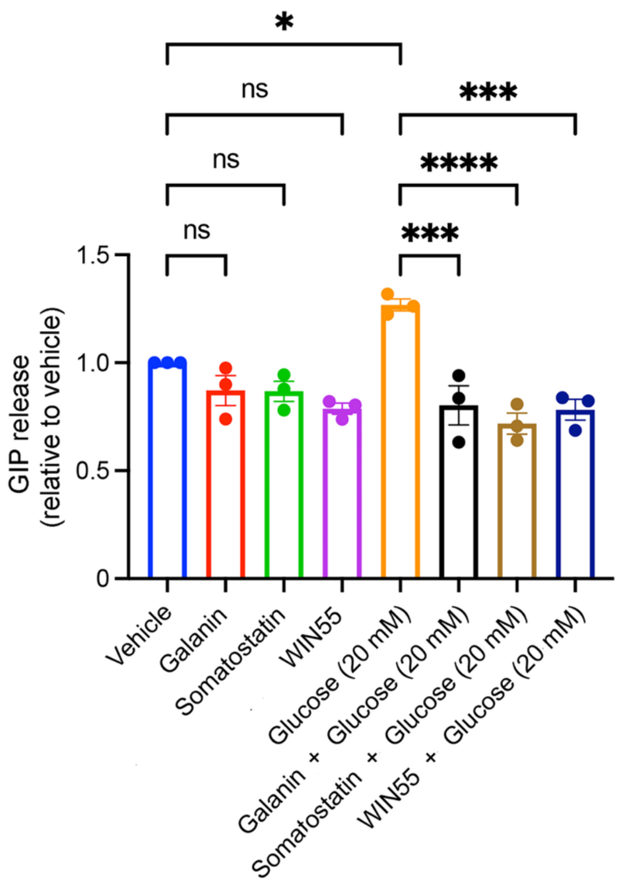

Fig. S8. Agonist activation of G_i-coupled receptors endogenously expressed by STC-1 cells. STC-1 cells were treated with galanin (100 nM), somatostatin (100 nM), or WIN55 (10 µM, positive control), followed by the measurement of GIP secretion into the medium. These agonists activate G_i-coupled receptors (galanin, somatostatin, and cannabinoid receptors, respectively) expressed by STC-1 cells. Data are given as means ± SEM from three independent experiments. GIP release was normalized relative to vehicle treatment (1 mM glucose). **P<0.05, ***P<0.001, ****P<0.0001 (1-way ANOVA followed by Tukey post-hoc analysis), as compared with the corresponding control group. ns, no statistically significant difference.

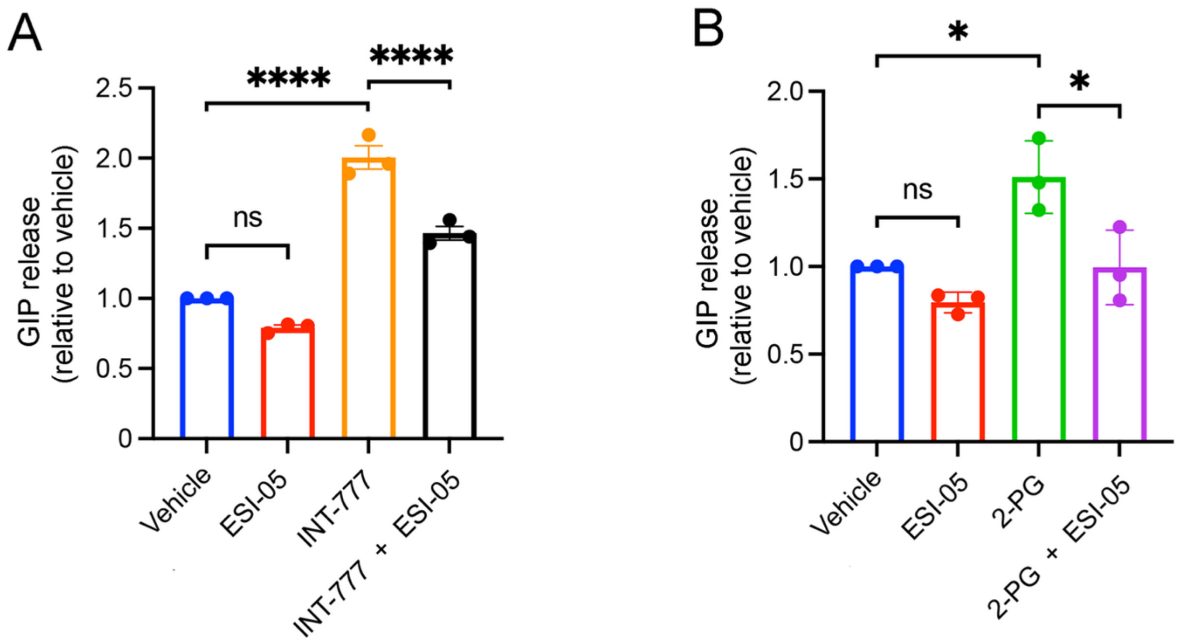

Fig. S9. Agonist activation of G$_s$-coupled receptors endogenously expressed by STC-1 cells. **(A, B)** Treatment of STC-1 cells with INT-777 (30 µM) (**A**), an agonist at the G$_s$-coupled GPBA receptor (former name: TGR5) or 2-palmitoyl glycerol (2-PG; 100 µM), an agonist at the G$_s$-coupled GPR119 receptor (**B**), stimulates GIP release. These stimulatory effects were greatly reduced by co-treatment of cells with a selective EPAC2 inhibitor (ESI-05, 10 µM) (**A, B**). Data are given as means ± SEM from three independent experiments. *P<0.05, ****P<0.0001 (1-way ANOVA followed by Tukey post-hoc analysis), as compared with the corresponding control group. ns, no statistically significant difference.

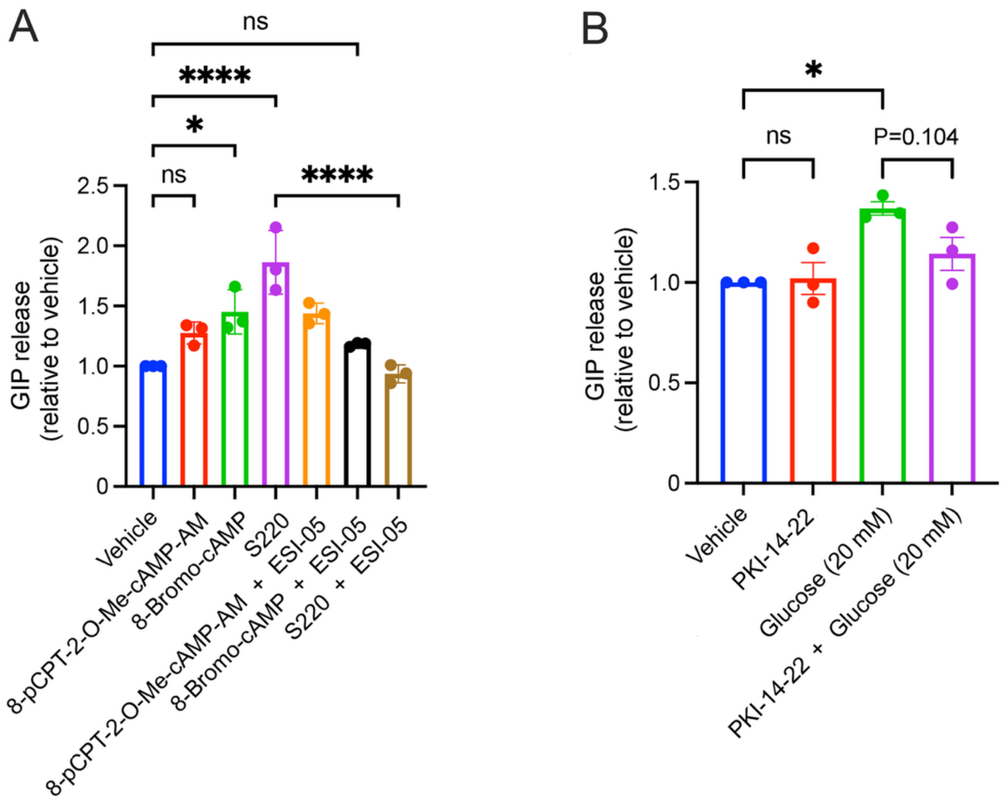

Fig. S10. Effect of EPAC and PKA activators on GIP release from STC-1 cells.

(A) STC-1 cells were treated with the following pharmacological agents, followed by the measurement of GIP secretion into the medium: 8-pCPT-2-O-Me-cAMP-AM (1 µM, selective EPAC1 activator), 8-bromo-cAMP (100 µM, selective PKA activator), and S220 (10 µM, selective EPAC2 activator; positive control). Note that treatment with the PKA and EPAC2 activators, but not with the EPAC1 activator, resulted in a significant stimulation of GIP secretion. **(B)** Treatment of STC-1 cells with a low concentration (100 nM) of PKI-14-22, a selective PKA inhibitor, led to a clear trend towards a reduction in glucose-stimulated GIP release ($P = 0.104$). Data are given as means ± SEM from three independent experiments. *$P<0.05$, ****$P<0.0001$ (1-way ANOVA followed by Tukey post-hoc analysis), as compared with the corresponding control group. ns, no statistically significant difference.

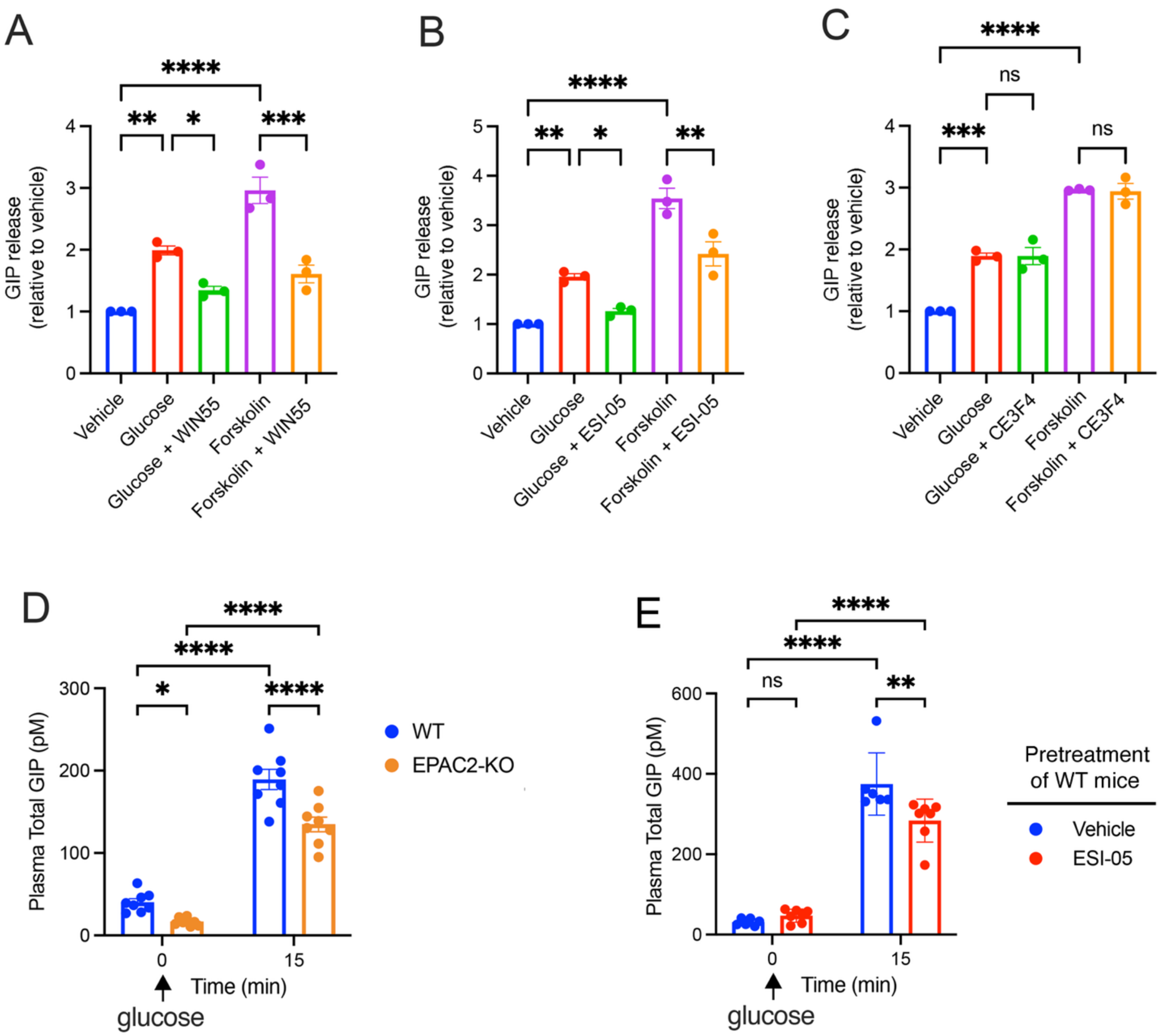

Fig. S11. **Glucose-stimulated GIP secretion studied with mouse duodenal organoids and WT and EPAC2 KO mice. (A-C)** Glucose-stimulated GIP release from mouse duodenal organoids in the presence of different pharmacologic agents. **(A, B)** The selective cannabinoid receptor agonist WIN55 (10 μM) (**A**) and the selective EPAC2 inhibitor ESI-05 (10 μM) (**B**) greatly reduced glucose (20 mM)-stimulated GIP release. (**C**) A selective EPAC1 inhibitor ((R)-CE3F4, 10 μM) had no significant effect on glucose-induced GIP secretion. Forskolin-stimulated GIP release served as an internal control in all experiments. Data are given as means ± SEM from three independent experiments. (**D**) Studies with EPAC2 KO mice. Following an oral

glucose bolus (2 g/kg), plasma GIP levels were determined in EPAC2 KO and WT control mice (8-week-old males). (**E**) Studies with WT mice pretreated with an EPAC2 inhibitor. WT mice (8-week-old C57/BL/6 male mice) were injected with the selective EPAC2 inhibitor ESI-05 (3 mg/kg i.p.). Thirty min later, the mice received an oral glucose bolus (2 g/kg). Plasma GIP levels were measured at the indicated time points. Data are given as means ± SEM (n = 8 mice/group). P<0.05, **P<0.01, ***P<0.01, ****P<0.0001 (1-way ANOVA (**A-C**) or 2-way ANOVA (**D, E**) followed by Tukey post-hoc analysis), as compared with the corresponding control group. ns, no statistically significant difference.

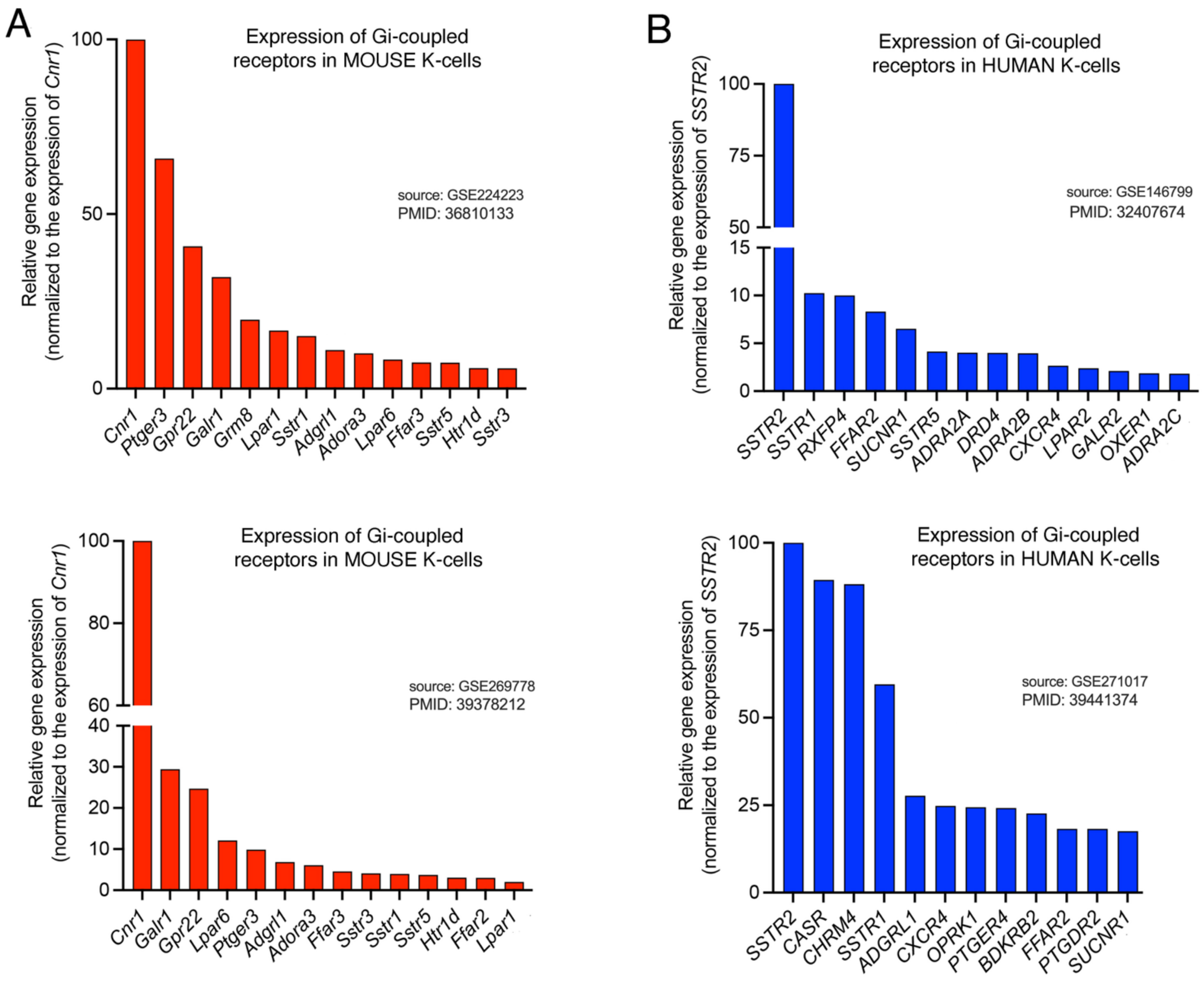

Fig. S12. Expression of mouse and human K-cell GPCRs that efficiently couple to G_i-type G proteins. (A) Mouse K-cells. Receptor gene expression levels were extracted from single-cell RNA sequencing (scRNA-seq) data from GIP-expressing mouse enteroendocrine cells. References: GSE224223 (53); GSE269778 (54). **(B)** Human K-cells. Receptor transcript levels were obtained from scRNA-seq data from GIP-expressing enteroendocrine cells contained within human duodenal/intestinal organoids. References: GSE146799 (72); GSE271017 (34).

Table S1. Primers used for mouse genotyping studies and gene expression analysis

Gene Target	Species	Primer Sequences
Gip-Cre	mouse	F-5’-GCTCCCTCCATTCACTTCACG<br>R-5’-GTAGTCCCTCACATCCTCAGG
Gi-DREADD (GiD)	mouse	F-5’-AAGGGAGCTGCAGTGGAGTA<br>R-5’-CCGAAAATCTGTGGGAAGTC
PTXRosa26	mouse	F-5’-AAAGTCGCTCTGAGTTGTTAT<br>R-5’-GCAGCGGGAGAAATGGATATG
Epac1 (Rapgef3)<br>(GEA)	mouse	F-5’-GCACCTACATCTGCAACAAGAGG<br>R-5’-CATCTCTGCTCACCAGGTCTGA
Epac2 (Rapgef4)<br>(GEA)	mouse	F-5’-CAATACGCCTTGAGCCATCGTTG<br>R-5’-CTTGAGAAGGCTGTGCGTGGTA
M36b4<br>(GEA)	mouse	F-5’-TCATCCAGCAGGTGTTTGACA<br>R-5’-GGCACCGAGGCAACAGTT
Gi-DREADD (GiD)<br>(GEA)	mouse	F-5’-TACGCTATGGCCAACTTCAC<br>R-5’-GACCATTTCCACCGTCTCATAG

GEA, primers used for gene expression analysis via qRT-PCR

REFERENCES

1. J. J. Holst, The incretin system in healthy humans: The role of GIP and GLP-1. *Metabolism* **96**, 46–55 (2019).

2. L. S. Gasbjerg, M. M. Helsted, B. Hartmann, M. H. Jensen, M. B. N. Gabe, A. H. Sparre-Ulrich, S. Veedfald, S. Stensen, A. R. Lanng, N. C. Bergmann, M. B. Christensen, T. Vilsbøll, J. J. Holst, M. M. Rosenkilde, F. K. Knop, Separate and combined glucometabolic effects of endogenous glucose-dependent insulinotropic polypeptide and glucagon-like peptide 1 in healthy individuals. *Diabetes* **68**, 906–917 (2019).

3. B. Ahrén, Glucose-dependent insulinotropic polypeptide secretion after oral macronutrient ingestion: The human literature revisited and a systematic study in model experiments in mice. *J. Diabet Investig.* **13**, 1655–1665 (2022).

4. T. D. Müller, A. Adriaenssens, B. Ahrén, M. Blüher, A. L. Birkenfeld, J. E. Campbell, M. P. Coghlan, D. D'Alessio, C. F. Deacon, S. DelPrato, J. D. Douros, D. J. Drucker, N. S. Figueredo Burgos, P. R. Flatt, B. Finan, R. E. Gimeno, F. M. Gribble, M. R. Hayes, C. Hölscher, J. J. Holst, P. J. Knerr, F. K. Knop, C. M. Kusminski, A. Liskiewicz, G. Mabilleau, S. A. Mowery, M. A. Nauck, A. Novikoff, F. Reimann, A. G. Roberts, M. M. Rosenkilde, R. J. Samms, P. E. Scherer, R. J. Seeley, K. W. Sloop, C. Wolfrum, D. Wootten, R. D. DiMarchi, M. H. Tschöp, Glucose-dependent insulinotropic polypeptide (GIP). *Mol. Metab.* **95**, 102118 (2025).

5. J. J. Holst, N. J. W. Albrechtsen, M. M. Rosenkilde, C. F. Deacon, Physiology of the incretin hormones, GIP and GLP-1-regulation of release and posttranslational modifications. *Compr. Physiol.* **9**, 1339–1381 (2019).

6. K. D. Galsgaard, J. Pedersen, F. K. Knop, J. J. Holst, N. J. Wewer Albrechtsen, Glucagon receptor signaling and lipid metabolism. *Front. Physiol.* **10**, 413 (2019).

7. N. Guccio, F. M. Gribble, F. Reimann, Glucose-dependent insulinotropic polypeptide—A postprandial hormone with unharnessed metabolic potential. *Annu Rev Nutrition* **42**, 21–44 (2022).

8. A. Nissen, M. Christensen, F. K. Knop, T. Vilsbøll, J. J. Holst, B. Hartmann, Glucose-dependent insulinotropic polypeptide inhibits bone resorption in humans. *J. Clin. Endocrinol. Metab.* **99**, E2325–E2329 (2014).

9. K. Skov-Jeppesen, C. B. Christiansen, L. S. Hansen, J. A. Windeløv, N. Hedbäck, L. S. Gasbjerg, M. Hindsø, M. S. Svane, S. Madsbad, J. J. Holst, M. M. Rosenkilde, B. Hartmann, Effects of exogenous GIP and GLP-2 on bone turnover in individuals with type 2 diabetes. *J. Clin. Endocrinol. Metab.* **109**, 1773–1780 (2024).

10. J. L. Beaudry, K. D. Kaur, E. M. Varin, L. L. Baggio, X. Cao, E. E. Mulvihill, H. E. Bates, J. E. Campbell, D. J. Drucker, Physiological roles of the GIP receptor in murine brown adipose tissue. *Mol. Metab.* **28**, 14–25 (2019).

11. R. Hammoud, K. D. Kaur, J. A. Koehler, L. L. Baggio, C. K. Wong, K. E. Advani, B. Yusta, I. Efimova, F. M. Gribble, F. Reimann, S. Fishman, C. Varol, D. J. Drucker, Glucose-dependent insulinotropic polypeptide receptor signaling alleviates gut inflammation in mice. *JCI Insight* **10**, e174825 (2024).

12. D. J. Drucker, Mechanisms of action and therapeutic application of glucagon-like peptide-1. *Cell Metab.* **27**, 740–756 (2018).

13. T. D. Muller, B. Finan, S. R. Bloom, D. D’Alessio, D. J. Drucker, P. R. Flatt, A. Fritsche, F. Gribble, H. J. Grill, J. F. Habener, J. J. Holst, W. Langhans, J. J. Meier, M. A. Nauck, D. Perez-Tilve, A. Pocai, F. Reimann, D. A. Sandoval, T. W. Schwartz, R. J. Seeley, K. Stemmer, M. Tang-Christensen, S. C. Woods, R. D. DiMarchi, M. H. Tschop, Glucagon-like peptide 1 (GLP-1). *Mol. Metab.* **30**, 72–130 (2019).

14. J. J. Holst, GLP-1 physiology in obesity and development of incretin-based drugs for chronic weight management. *Nat. Metab.* **6**, 1866–1885 (2024).

15. F. K. Knop, K. Aaboe, T. Vilsboll, A. Volund, J. J. Holst, T. Krarup, S. Madsbad, Impaired incretin effect and fasting hyperglucagonaemia characterizing type 2 diabetic subjects are early signs of dysmetabolism in obesity. *Diabetes Obes. Metab.* **14**, 500–510 (2012).

16. J. P. Frías, M. J. Davies, J. Rosenstock, F. C. Pérez Manghi, L. Fernández Landó, B. K. Bergman, B. Liu, X. Cui, K. Brown, Tirzepatide versus semaglutide once weekly in patients with type 2 diabetes. *New Engl J Med* **385**, 503–515 (2021).

17. T. Heise, A. Mari, J. H. DeVries, S. Urva, J. Li, E. J. Pratt, T. Coskun, M. K. Thomas, K. J. Mather, A. Haupt, Z. Milicevic, Effects of subcutaneous tirzepatide versus placebo or semaglutide on pancreatic islet function and insulin sensitivity in adults with type 2 diabetes: A multicentre, randomised, double-blind, parallel-arm, phase 1 clinical trial. *Lancet Diabet. Endocrinol.* **10**, 418–429 (2022).

18. A. M. Jastreboff, L. J. Aronne, N. N. Ahmad, S. Wharton, L. Connery, B. Alves, A. Kiyosue, S. Zhang, B. Liu, M. C. Bunck, A. Stefanski, Tirzepatide once weekly for the treatment of obesity. *New Engl J Med* **387**, 205–216 (2022).

19. T. Borner, A. M. Pataro, S. A. Doebley, C. D. Furst, A. D. White, S. X. Gao, A. Chow, M. J. Sanchez-Navarro, M. Y. Ghidewon, J. G. Halas, A. Z. Mohiby, F. S. Willard, H. J. Grill, M. Ai, R. J. Samms, M. R. Hayes, B. C. De Jonghe, Hypophagia and body weight loss by tirzepatide are accompanied by fewer GI adverse events compared to semaglutide in preclinical models. *Sci. Adv.* **11**, eadu1589 (2025).

20. B. Tan, X.-H. Pan, H. S. J. Chew, R. S. J. Goh, C. Lin, V. V. Anand, E. C. Z. Lee, K. E. Chan, G. Kong, C. E. Y. Ong, H. C. Chung, D. Y. Young, M. Y. Chan, C. M. Khoo, A. Mehta, M. D. Muthiah, M. Noureddin, C. H. Ng, N. W. S. Chew, Y. H. Chin, Efficacy and safety of tirzepatide for treatment of overweight or obesity. A systematic review and meta-analysis. *Int. J. Obes.* **47**, 677–685 (2005).

21. M. M. Rosenkilde, J. T. George, M. M. Véniant, J. J. Holst, GIP receptor antagonists in the pharmacotherapy of obesity: Physiologic, genetic, and clinical rationale. *Diabetes* **74**, 1334–1338 (2025).

22. F. Koefoed-Hansen, M. M. Helsted, H. S. Kizilkaya, A. B. Lund, M. M. Rosenkilde, L. S. Gasbjerg, The evolution of the therapeutic concept 'GIP receptor antagonism'. *Front. Endocrinol.* **16**, 1570603 (2025).

23. J. D. Douros, S. A. Mowery, P. J. Knerr, The premise of the paradox: Examining the evidence that motivated GIPR agonist and antagonist drug development programs. *J. Clin. Med.* **14**, 3812 (2025).

24. R. M. Gutgesell, A. Khalil, A. Liskiewicz, G. Maity-Kumar, A. Novikoff, G. Grandl, D. Liskiewicz, C. Coupland, E. Karaoglu, S. Akindehin, R. Castelino, F. Curion, X. Liu, C. Garcia-Caceres, A. Cebrian-Serrano, J. D. Douros, P. J. Knerr, B. Finan, R. D. DiMarchi, K. W. Sloop, R. J. Samms, F. J. Theis, M. H. Tschöp, T. D. Müller, GIPR agonism and antagonism decrease body weight and food intake via different mechanisms in male mice. *Nat. Metab.* **7**, 1282–1298 (2025).

25. A. E. Adriaenssens, Unravelling the GIPR agonist versus antagonist paradox. *Nat. Metab.* **7**, 1111–1113 (2025).

26. C. M. Liu, E. A. Killion, R. Hammoud, S. C. Lu, R. Komorowski, T. Liu, M. Kanke, V. A. Thomas, K. Cook, G. N. Sivits Jr., A. B. Ben, L. I. Atangan, R. Hussien, A. Tang, A. Shkumatov, C. M. Li, D. J. Drucker, M. M. Véniant, GIPR-Ab/GLP-1 peptide-antibody conjugate requires brain GIPR and GLP-1R for additive weight loss in obese mice. *Nat. Metab.* **7**, 1266–1281 (2025).

27. P. Larraufie, G. P. Roberts, A. K. McGavigan, R. G. Kay, J. Li, A. Leiter, A. Melvin, E. K. Biggs, P. Ravn, K. Davy, D. C. Hornigold, G. S. H. Yeo, R. H. Hardwick, F. Reimann, F. M. Gribble, Important role of the GLP-1 axis for glucose homeostasis after bariatric surgery. *Cell Rep.* **26**, 1399–1408.e6 (2019).

28. N. Çalık Başaran, I. Dotan, D. Dicker, Post metabolic bariatric surgery weight regain: The importance of GLP-1 levels. *Int. J. Obes.* **49**, 412–417 (2005).

29. M. Hindsø, N. Hedbäck, M. S. Svane, A. Møller, C. Martinussen, N. B. Jørgensen, C. Dirksen, L. S. Gasbjerg, V. B. Kristiansen, B. Hartmann, M. M. Rosenkilde, J. J. Holst, S. Madsbad, K. N. Bojsen-Møller, The importance of endogenously secreted GLP-1 and GIP for postprandial glucose tolerance and β-cell function after Roux-en-Y gastric bypass and sleeve gastrectomy surgery. *Diabetes* **72**, 336–347 (2023).

30. S. Andrade, C. B. Lobato, M. Machado, B. Hartmann, J. J. Holst, R. F. Almeida, M. Nora, M. P. Monteiro, M. Guimarães, S. S. Pereira, GLP-1 and GIP may play a role in long-term weight trajectories after gastric bypass. *Front. Endocrinol.* **16**, 1624001 (2025).

31. A. B. Oteng, L. Liu, Y. Cui, O. Gavrilova, H. Lu, M. Chen, L. S. Weinstein, J. E. Campbell, J. E. Lewis, F. M. Gribble, F. Reimann, J. Wess, Activation of G_s signaling in mouse enteroendocrine K cells greatly improves obesity- and diabetes-related metabolic deficits. *J. Clin. Invest.* **134**, e182325 (2024).

32. J. E. Lewis, D. Nuzzaci, P. P. James-Okoro, M. Montaner, E. O'Flaherty, T. Darwish, M. Hayashi, S. D. Liberles, D. Hornigold, J. Naylor, D. Baker, F. M. Gribble, F. Reimann, Stimulating intestinal GIP release reduces food intake and body weight in mice. *Mol. Metab.* **84**, 101945 (2024).

33. F. Reimann, G. Tolhurst, F. M. Gribble, G-protein-coupled receptors in intestinal chemosensation. *Cell Metab.* **15**, 421–431 (2012).

34. N. Guccio, C. Alcaino, E. L. Miedzybrodzka, M. Santos-Hernández, C. A. Smith, A. Davison, R. Bany Bakar, R. G. Kay, F. Reimann, F. M. Gribble, Molecular mechanisms underlying glucose-dependent insulinotropic polypeptide secretion in human duodenal organoids. *Diabetologia* **68**, 217–230 (2025).

35. K. Sriram, P. A. Insel, G protein-coupled receptors as targets for approved drugs: How many targets and how many drugs? *Mol. Pharmacol.* **93**, 251–258 (2018).

36. K. L. Pierce, R. T. Premont, R. J. Lefkowitz, Seven-transmembrane receptors. *Nat. Rev. Mol. Cell Biol.* **3**, 639–650 (2002).

37. N. Wettschureck, S. Offermanns, Mammalian G proteins and their cell type specific functions. *Physiol. Rev.* **85**, 1159–1204 (2005).

38. B. N. Armbruster, X. Li, M. H. Pausch, S. Herlitze, B. L. Roth, Evolving the lock to fit the key to create a family of G protein-coupled receptors potently activated by an inert ligand. *Proc. Natl. Acad. Sci. U.S.A.* **104**, 5163–5168 (2007).

39. D. J. Urban, B. L. Roth, DREADDs (designer receptors exclusively activated by designer drugs): Chemogenetic tools with therapeutic utility. *Annu. Rev. Pharmacol. Toxicol.* **55**, 399–417 (2015).

40. H. Zhu, D. K. Aryal, R. H. J. Olsen, D. J. Urban, A. Swearingen, S. Forbes, B. L. Roth, U. Hochgeschwender, Cre-dependent DREADD (designer receptors exclusively activated by designer drugs) mice. *Genesis* **54**, 439–446 (2016).

41. B. Svendsen, R. Pais, M. S. Engelstoft, N. B. Milev, P. Richards, C. B. Christiansen, K. L. Egerod, S. M. Jensen, A. M. Habib, F. M. Gribble, T. W. Schwartz, F. Reimann, J. J. Holst, GLP1- and GIP-producing cells rarely overlap and differ by bombesin receptor-2 expression and responsiveness. *J. Endocrinol.* **228**, 39–48 (2016).

42. J. B. Regard, H. Kataoka, D. A. Cano, E. Camerer, L. Yin, Y. W. Zheng, T. S. Scanlan, M. Hebrok, S. R. Coughlin, Probing cell type-specific functions of Gi in vivo identifies GPCR regulators of insulin secretion. *J. Clin. Invest.* **117**, 4034–4043 (2007).

43. H. Cheng, C. P. Leblond, Origin, differentiation and renewal of the four main epithelial cell types in the mouse small intestine. V. Unitarian Theory of the origin of the four epithelial cell types. *Am. J. Anat.* **141**, 537–561 (1974).

44. Y. Nagai, N. Miyakawa, H. Takuwa, Y. Hori, K. Oyama, B. Ji, M. Takahashi, X. P. Huang, S. T. Slocum, J. F. DiBerto, Y. Xiong, T. Urushihata, T. Hirabayashi, A. Fujimoto, K. Mimura, J. G. English, J. Liu, K. I. Inoue, K. Kumata, C. Seki, M. Ono, M. Shimojo, M. R. Zhang, Y. Tomita, J. Nakahara, T. Suhara, M. Takada, M. Higuchi, J. Jin, B. L. Roth, T. Minamimoto, Deschloroclozapine, a potent and selective chemogenetic actuator enables rapid neuronal and behavioral modulations in mice and monkeys. *Nat. Neurosci* **23**, 1157–1167 (2020).

45. M. Santos-Hernández, F. Reimann, F. M. Gribble, Cellular mechanisms of incretin hormone secretion. *J. Mol. Endocrinol.* **72**, e230112 (2024).

46. S. P. H. Alexander, A. Christopoulos, A. P. Davenport, E. Kelly, A. A. Mathie, J. A. Peters, E. L. Veale, J. F. Armstrong, E. Faccenda, S. D. Harding, J. A. Davies, M. P. Abbracchio, G. Abraham, A. Agoulnik, W. Alexander, K. Al-Hosaini, M. Bäck, J. G. Baker, N. M. Barnes, R.

Bathgate, J. M. Beaulieu, A. G. Beck-Sickinger, M. Behrens, K. E. Bernstein, B. Bettler, N. J. M. Birdsall, V. Blaho, F. Boulay, C. Bousquet, H. Bräuner-Osborne, G. Burnstock, G. Caló, J. P. Castaño, K. J. Catt, S. Ceruti, P. Chazot, N. Chiang, B. Chini, J. Chun, A. Cianciulli, O. Civelli, L. H. Clapp, R. Couture, H. M. Cox, Z. Csaba, C. Dahlgren, G. Dent, S. D. Douglas, P. D. S. Eguchi, E. Escher, E. J. Filardo, T. Fong, M. Fumagalli, R. R. Gainetdinov, M. L. Garelja, M. de Gasparo, C. Gerard, M. Gershengorn, F. Gobeil, T. L. Goodfriend, C. Goudet, L. Grätz, K. J. Gregory, A. L. Gundlach, J. Hamann, J. Hanson, R. L. Hauger, D. L. Hay, A. Heinemann, D. Herr, M. D. Hollenberg, N. D. Holliday, M. Horiuchi, D. Hoyer, L. Hunyady, A. Husain, A. P. IJzerman, T. Inagami, K. A. Jacobson, R. T. Jensen, R. Jockers, D. Jonnalagadda, S. Karnik, K. Kaupmann, J. Kemp, C. Kennedy, Y. Kihara, T. Kitazawa, P. Kozielewicz, H.-J. Kreienkamp, J. P. Kukkonen, T. Langenhan, D. Larhammar, K. Leach, D. Lecca, J. D. Lee, S. E. Leeman, J. Leprince, X. X. Li, S. J. Lolait, A. Lupp, R. Macrae, J. Maguire, D. Malfacini, J. Mazella, C. A. M. Ardle, S. Melmed, M. C. Michel, L. J. Miller, V. Mitolo, B. Mouillac, C. E. Müller, P. M. Murphy, J.-L. Nahon, T. Ngo, X. Norel, D. Nyimanu, A.-M. O'Carroll, S. Offermanns, M. A. Panaro, M. Parmentier, R. G. Pertwee, J.-P. Pin, E. R. Prossnitz, M. Quinn, R. Ramachandran, M. Ray, R. K. Reinscheid, P. Rondard, G. E. Rovati, C. Ruzza, G. J. Sanger, T. Schöneberg, G. Schulte, S. Schulz, D. L. Segaloff, C. N. Serhan, K. D. Singh, C. M. Smith, L. A. Stoddart, Y. Sugimoto, R. Summers, V. P. Tan, D. Thal, W. W. Thomas, P. B. M. W. M. Timmermans, K. Tirupula, L. Toll, G. Tulipano, H. Unal, T. Unger, C. Valant, P. Vanderheyden, D. Vaudry, H. Vaudry, J.-P. Vilardaga, C. S. Walker, J. M. Wang, D. T. Ward, H.-J. Wester, G. B. Willars, T. L. Williams, T. M. Woodruff, C. Yao, R. D. Ye, The concise guide to PHARMACOLOGY 2023/24: G protein-coupled receptors. *Br. J. Pharmacol.* **180**, S23–S144 (2023).

47. L. Wang, S. P. Pydi, L. Zhu, L. F. Barella, Y. Cui, O. Gavrilova, K. K. Bence, C. Vernochet, J. Wess, Adipocyte G_i signaling is essential for maintaining whole-body glucose homeostasis and insulin sensitivity. *Nat. Commun.* **11**, 2995 (2020).

48. M. Rossi, L. Zhu, S. M. McMillin, S. P. Pydi, S. Jain, L. Wang, Y. Cui, R. J. Lee, A. H. Cohen, H. Kaneto, M. J. Birnbaum, Y. Ma, Y. Rotman, J. Liu, T. J. Cyphert, T. Finkel, O. P. McGuinness, J. Wess, Hepatic G_i signaling regulates whole-body glucose homeostasis. *J. Clin. Invest.* **128**, 746–759 (2018).

49. J. A. West, A. Tsakmaki, S. S. Ghosh, D. G. Parkes, R. V. Grønlund, P. J. Pedersen, D. Maggs, H. Rajagopalan, G. A. Bewick, Chronic peptide-based GIP receptor inhibition exhibits modest glucose metabolic changes in mice when administered either alone or combined with GLP-1 agonism. *PLOS ONE* **16**, e0249239 (2021).

50. S. Jain, I. Ruiz de Azua, H. Lu, M. F. White, J. M. Guettier, J. Wess, Chronic activation of a designer G_q-coupled receptor improves β cell function. *J. Clin. Invest.* **123**, 1750–1762 (2013).

51. A. Maida, T. Hansotia, C. Longuet, Y. Seino, D. J. Drucker, Differential importance of glucose-dependent insulinotropic polypeptide vs glucagon-like peptide 1 receptor signaling for beta cell survival in mice. *Gastroenterology* **137**, 2146–2157 (2009).

52. T. McCarthy, B. D. Green, D. Calderwood, A. Gillespie, J. F. Cryan, L. Giblin, "STC-1 cells," in *The Impact of Food Bioactives on Health: In vitro and ex vivo models*, K. Verhoeckx, P. Cotter, I. López-Expósito, C. Kleiveland, T. Lea, A. Mackie, T. Requena, D. Swiatecka, H. Wichers, Eds. (Springer, 2015), pp. 211–220.

53. M. Hayashi, J. A. Kaye, E. R. Douglas, N. R. Joshi, F. M. Gribble, F. Reimann, S. D. Liberles, Enteroendocrine cell lineages that differentially control feeding and gut motility. *ELife* **12**, e78512 (2023).

54. C. A. Smith, E. A. A. O'Flaherty, N. Guccio, A. Punnoose, T. Darwish, J. E. Lewis, R. E. Foreman, J. Li, R. G. Kay, A. E. Adriaenssens, F. Reimann, F. M. Gribble, Single-cell transcriptomic atlas of enteroendocrine cells along the murine gastrointestinal tract. *PLOS ONE* **19**, e0308942 (2024).

55. D. Hodge, W. J. Marsh, H. E. Parker, E. Ogunnowo-Bada, C. H. Riches, A. M. Habib, M. L. Evans, F. M. Gribble, F. Reimann, Somatostatin receptor 5 and cannabinoid receptor 1 activation inhibit secretion of glucose-dependent insulinotropic polypeptide from intestinal K cells in rodents. *Diabetologia* **55**, 3094–3103 (2012).

56. L. M. Casey, A. R. Pistner, S. L. Belmonte, D. Migdalovich, O. Stolpnik, F. E. Nwakanma, G. Vorobiof, O. Dunaevsky, A. Matavel, C. M. Lopes, A. V. Smrcka, B. C. Blaxall, Small

molecule disruption of G beta gamma signaling inhibits the progression of heart failure. *Circ. Res.* **107**, 532–539 (2010).

57. M. Gloerich, J. L. Bos, Epac: Defining a new mechanism for cAMP action. *Annu. Rev. Pharmacol. Toxicol.* **50**, 355–375 (2010).

58. F. Schwede, D. Bertinetti, C. N. Langerijs, M. A. Hadders, H. Wienk, J. H. Ellenbroek, E. J. de Koning, J. L. Bos, F. W. Herberg, H. G. Genieser, R. A. Janssen, H. Rehmann, Structure-guided design of selective Epac1 and Epac2 agonists. *PLoS Biol.* **13**, e1002038 (2015).

59. A. Psichas, L. L. Glass, S. J. Sharp, F. Reimann, F. M. Gribble, Galanin inhibits GLP-1 and GIP secretion via the GAL1 receptor in enteroendocrine L and K cells. *Br. J. Pharmacol.* **173**, 888–898 (2016).

60. H. Zhu, K. Wang, S. Chen, J. Kang, N. Guo, H. Chen, J. Liu, Y. Wu, P. He, Y. Tu, B. Li, Saponins from *Camellia sinensis* seeds stimulate GIP secretion in mice and STC-1 cells via SGLT1 and TGR5. *Nutrients* **14**, 3413 (2022).

61. Y. Funatsu, N. Takahashi, Y. Yamazaki, H. Watanabe, T. Nakagita, Y. Ishimaru, K. Nakamura, K. Kaneko, Fatty acid composition in 2-monoacylglycerol modulates GLP-1 secretion. *Biochem. Biophys. Res. Commun.* **781**, 152546 (2025).

62. J. J. Holst, M. M. Rosenkilde, GIP as a therapeutic target in diabetes and obesity: Insight from incretin co-agonists. *J. Clin. Endocrinol. Metab.* **105**, e2710–e2716 (2020).

63. C. Hasenoehrl, U. Taschler, M. Storr, R. Schicho, The gastrointestinal tract – A central organ of cannabinoid signaling in health and disease. *Neurogastroenterol. Motil.* **28**, 1765–1780 (2016).

64. X. Cheng, Z. Ji, T. Tsalkova, F. Mei, Epac and PKA: A tale of two intracellular cAMP receptors. *Acta Biochim. Biophys. Sin.* **40**, 651–662 (2008).

65. T. Shibasaki, H. Takahashi, T. Miki, Y. Sunaga, K. Matsumura, M. Yamanaka, C. Zhang, A. Tamamoto, T. Satoh, J. Miyazaki, S. Seino, Essential role of Epac2/Rap1 signaling in

regulation of insulin granule dynamics by cAMP. *Proc. Natl. Acad. Sci. U.S.A.* **104**, 19333–19338 (2007).

66. I. Dzhura, O. G. Chepurny, C. A. Leech, M. W. Roe, E. Dzhura, X. Xu, Y. Lu, F. Schwede, H. G. Genieser, A. V. Smrcka, G. G. Holz, Phospholipase C-ε links Epac2 activation to the potentiation of glucose-stimulated insulin secretion from mouse islets of Langerhans. *Islets* **3**, 121–128 (2011).

67. H. S. Hansen, M. M. Rosenkilde, J. J. Holst, T. W. Schwartz, GPR119 as a fat sensor. *Trends Pharmacol. Sci.* **33**, 374–381 (2012).

68. M. M. Smits, K. D. Galsgaard, S. L. Jepsen, N. W. Albrechtsen, B. Hartmann, J. J. Holst, In vivo inhibition of dipeptidyl peptidase 4 allows measurement of GLP-1 secretion in mice. *Diabetes* **73**, 671–681 (2024).

69. I. K. Sebhat, M. J. M. Murphy, S. Zheng, R. J. Lovelett, M. Engelstoft, D. Kosinski, X. Yang, V. Dunn, J. Whang, M. G. Lombardo, A. Heilbut, G. Terracina, N. Nicholas, M. Leitner, M. J. Consolati, B. Chan, G. Poterewicz, A. Vance, J. Liu, A. E. Weber, B. Lauring, N. Thornberry, S. Pinto, Gut enteroendocrine cell activation using a combination of GPR119 and GPR40 agonists results in synergistic hormone secretion in mice and humans. *Cell Metab.* **38**, 50–64.e12 (2026).

70. L. Pereira, H. Cheng, D. H. Lao, L. Na, R. J. van Oort, J. H. Brown, X. H. Wehrens, J. Chen, D. M. Bers, Epac2 mediates cardiac β1-adrenergic-dependent sarcoplasmic reticulum Ca^{2+} leak and arrhythmia. *Circulation* **127**, 913–922 (2013).

71. M. M. Mahe, E. Aihara, M. A. Schumacher, Y. Zavros, M. H. Montrose, M. A. Helmrath, T. Sato, N. F. Shroyer, Establishment of gastrointestinal epithelial organoids. *Curr. Protoc. Mouse Biol.* **3**, 217–240 (2013).

72. J. Beumer, J. Puschhof, J. Bauzá-Martinez, A. Martínez-Silgado, R. Elmentaite, K. R. James, A. Ross, D. Hendriks, B. Artegiani, G. A. Busslinger, B. Ponsioen, A. Andersson-Rolf, A. Saftien, C. Boot, K. Kretzschmar, M. H. Geurts, Y. E. Bar-Ephraim, C. Pleguezuelos-Manzano, Y. Post, H. Begthel, F. van der Linden, C. Lopez-Iglesias, W. J. van de Wetering,

R. van der Linden, P. J. Peters, A. J. R. Heck, J. Goedhart, H. Snippert, M. Zilbauer, S. A. Teichmann, W. Wu, H. Clevers, High-resolution mRNA and secretome atlas of human enteroendocrine cells. *Cell* **182**, 1062–1064 (2020).
